# Manipulating Charge Dynamics in Carbon Nitride by Carbon Dot Doping for Efficient Photocatalysis

**DOI:** 10.1002/advs.202417390

**Published:** 2025-04-26

**Authors:** Lingfeng Ouyang, Maggie Ng, Zhang‐Hong Zhou, Hao Wu, Man‐Chung Tang, Season Si Chen

**Affiliations:** ^1^ Institute of Environment and Ecology Tsinghua Shenzhen International Graduate School Tsinghua University Shenzhen 518005 P. R. China; ^2^ Macau Institute of Materials Science and Engineering (MIMSE) Faculty of Innovation Engineering Macau University of Science and Technology Taipa Macau 999078 P. R. China; ^3^ Institute of Materials Research Tsinghua Shenzhen International Graduate School Tsinghua University Shenzhen 518005 P. R. China

**Keywords:** carbon dots, charge transfer excited state, emerging contaminants, graphitic carbon nitride, photocatalysis

## Abstract

Graphitic carbon nitride (g‐C_3_N_4_), a prominent metal‐free semiconductor photocatalyst, faces limitations due to its high exciton binding energy. While significant efforts have been focused on optimizing charge‐carrier processes, the interplay of exciton and free carrier in this system have received less attention. Herein, this density‐functional theory (DFT) and time‐dependent DFT calculations demonstrate that carbon dot‐functionalized g‐C_3_N_4_ (g‐C_3_N_4_/CD), synthesized via a facile thermal polymerization, shifts the excited state from localized to charge transfer characteristics. The g‐C_3_N_4_/CD exhibits reduced exciton binding energy from 41.0 to 24.6 meV, as shown by temperature‐dependent photoluminescence spectroscopy. Particularly, g‐C_3_N_4_/CD‐10 (10 wt.% CD solution in precursors) achieves a 3‐fold increase in the photodegradation rate (*k* = 0.020 min⁻¹) of an emerging environmental pollutant, levofloxacin (LEV), under 10 W LED light. Enhanced photocatalytic performances correlate with optimized band structure and efficient charge transport, as confirmed by photophysical and photoelectrochemical analyses. Although the excited state lifetime in g‐C_3_N_4_/CD is slightly reduced compared to pristine g‐C_3_N_4_, photocatalytic activity remains unaffected, underscoring the critical role of charge excited state in enhancing photocatalytic efficiency. This work offers insights onto the potential of manipulating charge transfer excited state dynamics for improved g‐C_3_N_4_‐based photocatalysis in environmental applications.

## Graphical abstract

A series of carbon dot (CD) doped graphitic carbon nitride (g‐C_3_N_4_) materials have been rationally synthesized, which exhibited different photophysical properties, excitonic behaviors, and photocatalytic degradation activities. It provides valuable insights to deepen the understanding of exciton dynamics based on g‐C_3_N_4_ materials, and rational design of high‐efficiency photocatalysts.

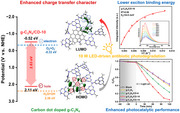

## 1 Introduction

The advent and overuse of antibiotics in healthcare and agriculture, inadequate waste management, and the subsequent environmental transmission have led to their widespread detection in various environmental matrices.^[^ ^1^ ^]^ Notably, fluoroquinolones, a class of antibiotics, render more inherent resistance to degradation compared to other antibiotic categories owing to the presence of robust C─F bonds (with a bond dissociation energy of ≈111 kcal mol^−1^).^[^ ^2^ ^]^ As a result, fluoroquinolones have become the most frequently found antibiotics in various water bodies (e.g., river, domestic sewage, groundwater, and hospital effluent) at concentration levels ranging from µg L^−1^ to mg L^−1^,^[^ ^3^ ^]^ which has exacerbated antimicrobial resistance and associated bacterial persistence that threaten the public health and the ecological environment.^[^ ^4^ ^]^ Various advanced oxidation processes such as Fenton processes, ozonation, electrochemical oxidation, and plasma‐based processes have proven effective for a wide range of antibiotic removal.^[^ ^5^ ^]^ Among these, solar‐driven photocatalysis has emerged as a green and sustainable approach, leveraging the renewable source of sunlight and the ease of heterogeneous catalyst recycling.^[^ ^6^ ^]^ In particular, metal‐free and carbon‐based photocatalysts offer distinct advantages, including visible light absorption, environmental friendliness, biocompatibility, and high stability.^[^ ^7^ ^]^

Graphitic carbon nitride (g‐C_3_N_4_) is an emerging group of carbon‐based and metal‐free semiconductors and has demonstrated favorable characteristics in photocatalysis owing to its suitable band gap (2.7−2.9 eV), ease of synthesis, facile structure modification, and intrinsic stability.^[^ ^8^ ^]^ However, the low dielectric property of its tris‐s‐triazine‐based network^[^ ^9^ ^]^ results in strong Coulombic interactions between photogenerated electron and hole pairs, rendering a localized excited (LE) state and rapid charge recombination via both radiative and nonradiative pathways.^[^ ^10^ ^]^ Current research aimed at enhancing the photocatalytic activity of g‐C_3_N_4_ mainly focuses on optimizing the charge‐carrier processes based on band theory. Effective strategies include band structure adjustments and charge‐carrier behavior optimization through heteroatom doping, heterojunction formation, and interfacial junction.^[^ ^11^ ^]^ Recently, various techniques have been reported to functionalize g‐C_3_N_4_ with carbon dots (CD), emerging low‐cost carbonaceous nanomaterials known for their flexible electron donating and withdrawing capabilities, to improve the separation of photoexcited carriers.^[^ ^12^ ^]^ While the role of heterostructures in the g‐C_3_N_4_/CD photocatalytic system has been well‐documented, the contributions of another photogenerated species, exciton (bound electron‐hole pairs), have received less attention. Given the strong exitonic effects in the g‐C_3_N_4_ matrix, excitons can dominate as the primary photoinduced species.^[^ ^13^ ^]^

Considering the competition between charge carrier generation and exciton generation, it is crucial to explore exciton processes, as they may significantly supplement carrier processes. Therefore, studying exciton‐involved photophysical processes in g‐C_3_N_4_‐based photocatalytic systems is essential for achieving high‐efficiency photocatalytic applications. In this context, we utilized g‐C_3_N_4_/CD systems as a model for investigation. Density‐functional theory (DFT) and time‐dependent DFT (TD‐DFT) calculations revealed that doping with CD dramatically altered the electron distribution of pristine g‐C_3_N_4_ molecule, endowing it with charge transfer (CT) excited state characteristics. The perturbance of excited state character led to a reduction in exciton binding energy (*E*_b_), as demonstrated by temperature‐dependent photoluminescence spectroscopy. The decrease *E*_b_ in the g‐C_3_N_4_/CD systems facilitated the dissociation of excitons into free charge carriers, thereby enhancing charge separation efficiency compared to pristine g‐C_3_N_4_, as confirmed by photoelectrochemical measurements.

In the photocatalytic tests, g‐C_3_N_4_/CD‐10 (10 wt% addition of CD solution prior to nanocomposite synthesis) exhibited over 95% removal efficiency of levofloxacin (LEV) under LED irradiation. The practical applicability of g‐C_3_N_4_/CD‐10 was demonstrated through its efficacy and stability in recycle tests, as well as its performance in treating complex water matrices, including varying pH solutions, perturbance of common ions and natural organic matter in real water samples (e.g., tap water, lake water, river water and sewage water), highlighting its potential for environmental remediation applications. This study emphasizes the critical role of excited‐state properties in augmenting the photocatalytic performance of g‐C_3_N_4_/CD‐based materials, presenting a feasible approach to enhance the efficiency of photocatalytic systems for antibiotic degradation in real‐world scenarios.

## 2 Results and Discussion

**Figure 1:**
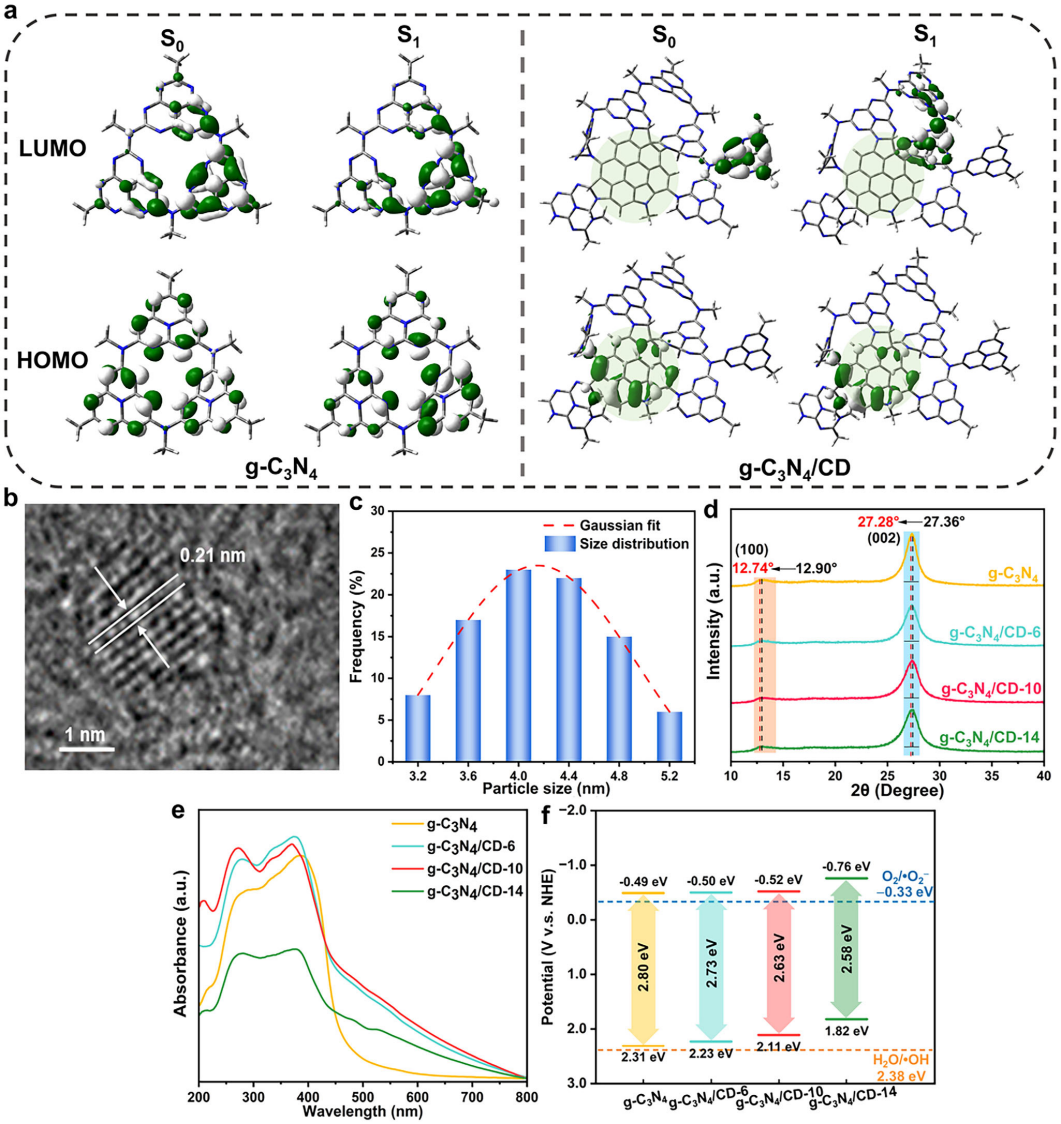
a) Spatial distributions of the HOMO‐LUMO orbitals of g‐C_3_N_4_ and g‐C_3_N_4_/CD at the S_0_ and S_1_ geometries (The green circle area represents the CD electronic structure). (a) Spatial distributions of the HOMO‐LUMO orbitals of g‐C_3_N_4_ and g‐C_3_N_4_/CD at the S_0_ and S_1_ geometries. b) HRTEM image, c) particle size distribution of CD in aqueous solutions. d) XRD patterns, e) UV–vis DRS spectra, and f) band gaps and structures of g‐C_3_N_4_, g‐C_3_N_4_/CD‐6, g‐C_3_N_4_/CD‐10, and g‐C_3_N_4_/CD‐14.

We first performed DFT and TD‐DFT calculations to investigate the electronic structure on both the S_0_ and S_1_ states of g‐C_3_N_4_ and g‐C_3_N_4_/CD, and the optimized CD atomic model is shown in Figure S1 (Supporting Information). The HOMO and LUMO of g‐C_3_N_4_ and g‐C_3_N_4_/CD are illustrated in **Figure** **1**a as the examples. The S_0_ and S_1_ states of g‐C_3_N_4_ were found to be localized on the g‐C_3_N_4_ backbone, indicating the predominant localized excited (LE) character of the S_1_ state. In contrast, the HOMO for both the S_0_ and S_1_ states of g‐C_3_N_4_/CD was localized on the CD moiety, whereas the LUMO was localized on the g‐C_3_N_4_ moiety, suggesting a charge transfer (CT) character of the S_1_ state of g‐C_3_N_4_/CD, with an intrinsic CT percentage of 51% based on neutral transition orbital calculations. The excited state characteristics were further validated by calculating the *S*_r_/D values, where *S*_r_ represents the overlap of hole‐electron distribution and D is the hole‐electron separation distance (Table S1, Supporting Information). A smaller *S*_r_ value accompanied by a larger D value signifies a more pronounced charge transfer.^[^ ^14^ ^]^ The calculated *S*_r_ and D values for pristine g‐C_3_N_4_ are 0.463 and 0.166 Å, respectively (*S*_r_/D *=* 2.789 Å^−1^), whereas the g‐C_3_N_4_/CD composite exhibits a reduced *S*_r_/D ratio of 0.213 Å^−1^ (*S*_r_ = 0.691, D = 3.242 Å), corroborating the excited assignments. These results align with the negative *t*‐index of g‐C_3_N_4_ (−1.07 Å; LE character) and positive value in g‐C_3_N_4_/CD (0.38 Å, CT character), as well as their electrostatic potential analyses (Figure S2, Supporting Information).^[^ ^15^ ^]^

The design and synthesis of CD, g‐C_3_N_4_, and g‐C_3_N_4_/CD‐x (“x” represents the mass doping percentages of CD) have been confirmed by comprehensive morphology imaging, structural characterization, and optical properties. High‐resolution TEM (HRTEM) imaging revealed the presence of CD on the surface of the g‐C₃N₄ matrix as darker spots (Figures S3a–c, Supporting Information). The CD appeared as uniformly dispersed, ellipsoidal particles with a narrow size range of 3.38–5.27 nm (average 4.19 ± 0.60 nm) and a lattice spacing of 0.21 nm, characteristic of graphite (100) planes (Figures 1b&c).^[^ ^16^ ^]^ Notably, no aggregation of CD was observed, indicating their effective dispersion within the composite. The XRD patterns (Figure 1d) confirmed the preservation of the characteristic g‐C_3_N_4_ peaks at 2θ values of ≈12.90° and 27.36°, corresponding to the (100) and (002) planes, respectively. Furthermore, the progressive shift of the (002) peak from 27.36° to 27.28° with increasing CD doping suggests an expanded interlayer spacing, indicative of the successful intercalation of CD into the g‐C₃N₄ matrix.^[^ ^17^ ^]^ The UV–vis absorption spectra showed that the g‐C_3_N_4_/CD samples exhibited enhanced visible light absorption and a significant redshift compared to pristine g‐C_3_N_4_ (Figure 1e). The color of the samples changed from light yellow to dark brown with increasing CD content (Figure S4, Supporting Information), suggesting the altered energy band structure and broadened visible light response upon CD doping. The red shift of the absorption spectra of g‐C_3_N_4_/CD with increasing CD doping reflected the electron‐doping effect of CD, which altered the electronic energy levels of the systems. Specifically, the increase in the doping concentration of CD in the doped system destabilizes the HOMO energy level to a greater extent than the destabilization of the LUMO energy level, resulting in a narrower HOMO‐LUMO energy gap. The band gap energies (*E*_g_) calculated from Tauc plots (Figure S5a, Supporting Information) gradually decreased from 2.80 to 2.58 eV with the addition of CD from g‐C_3_N_4_ to g‐C_3_N_4_/CD‐14, suggesting improved visible light harvesting. Furthermore, the valence band maximum (VBM) measured by VB‐XPS (Figure S5b, Supporting Information) decreased from 2.31 eV (g‐C_3_N_4_) to 1.82 eV (g‐C_3_N_4_/CD‐14), while the conduction band minimum (CBM) shifted from −0.49 eV (g‐C_3_N_4_) to −0.76 eV (g‐C_3_N_4_/CD‐14) given the formula that *E*_VB_ = *E*_CB_ + *E*_g_. The band structures of all samples were illustrated in Figure 1f.

**Figure 2:**
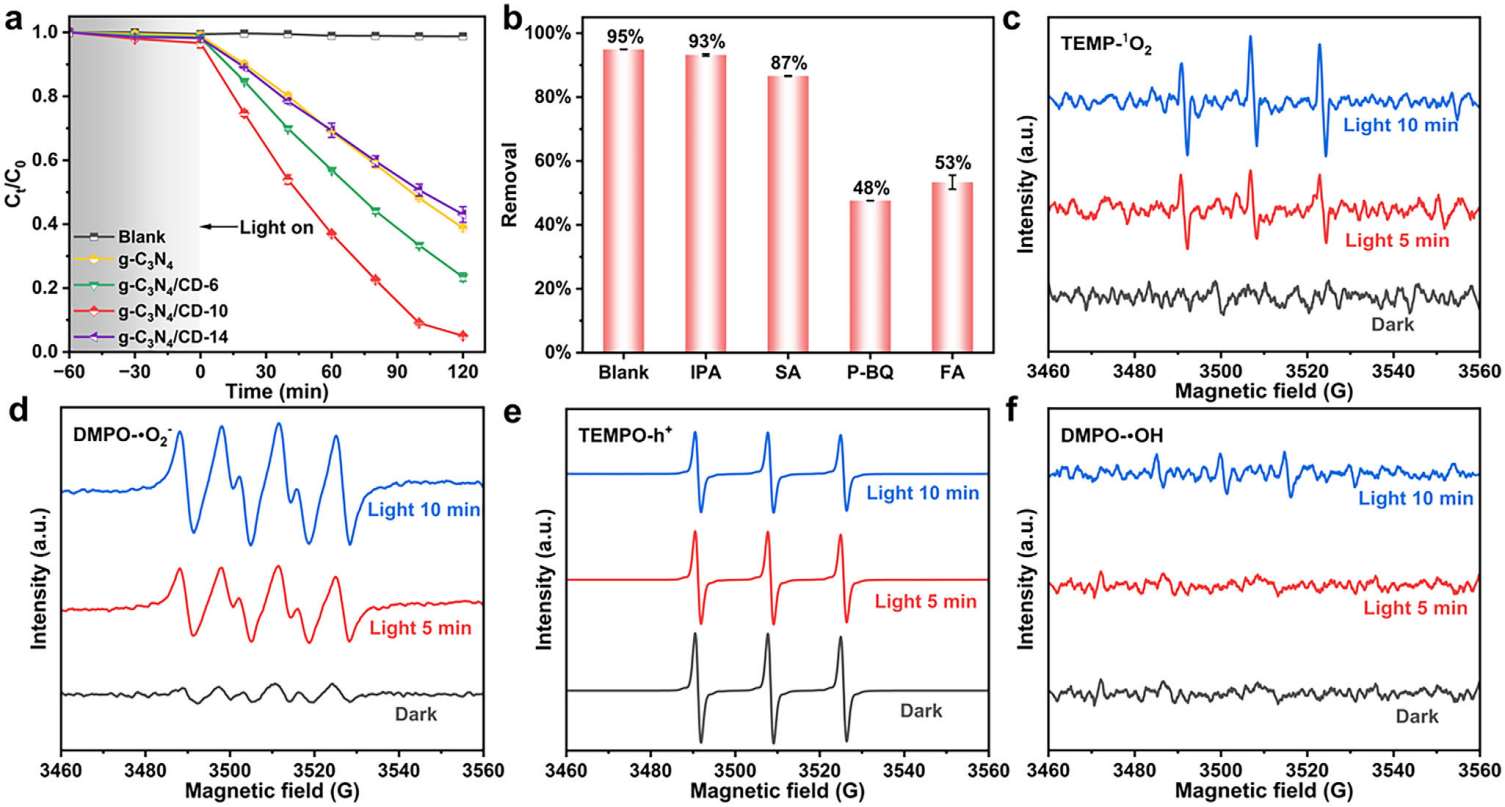
a) Photocatalytic activity for LEV degradation by different photocatalysts under LED illumination (Reaction conditions: [LEV] = 10 ppm, [photocatalyst] = 0.5 g L^−1^, Initial pH = 7.0, T = 25 °C, Reaction time = 2 h, Pre‐adsorption time = 1 h), b) Photocatalytic performance of g‐C_3_N_4_/CD‐10 with different scavengers under identical photoreaction conditions. ESR signals of g‐C_3_N_4_/CD‐10 photocatalyst for c) ^1^O_2_, d) •O_2_^−^, (e) h^+^, and (f) •OH−.

The photocatalytic degradation efficiencies of g‐C_3_N_4_ and g‐C_3_N_4_/CD were evaluated with 10 mg L^−1^ LEV solutions. Under dark conditions upon adsorption equilibrium, negligible LEV was adsorbed onto the photocatalysts (**Figure** **2**a). In the absence of a photocatalyst, LED light illumination induced no photolysis of LEV. Among the tested materials, the g‐C_3_N_4_/CD photocatalysts outperformed pristine g‐C_3_N_4_ in their photocatalytic capabilities for LEV degradation, except for g‐C_3_N_4_/CD‐14, which showed comparable performance to g‐C_3_N_4_ at ≈60% removal. The LEV removal rates ranged from the lowest at ∼60% for both g‐C_3_N_4_ and g‐C_3_N_4_/CD‐14, to 80% for g‐C_3_N_4_/CD‐6, and the highest over 95% for g‐C_3_N_4_/CD‐10. Specifically, LEV removal exhibited the highest apparent rate constant of 2 × 10^−2^ min^−1^ over g‐C_3_N_4_/CD‐10, marking a ca. 3‐fold enhancement compared to that of g‐C_3_N_4_ (0.7 × 10^−2^ min^−1^) (Figure S6a, Supporting Information). The outlier of g‐C_3_N_4_/CD‐14 was partially attributed to the upward shift of valence band maximum compared to those in other photocatalyst samples, indicating a weak oxidation power of holes generated on the valence band and thus hindered photooxidation efficiency of LEV (Figure 1f).^[^ ^18^ ^]^ These results emphasized the importance of band structure in photocatalytic performance, in addition to the exciton dissociation and charge transfer processes discussed in the latter part of this work. Notably, g‐C_3_N_4_/CD‐10 exhibited a leading equivalent environmental efficiency of 95.0 mg (mg·kW·h)^−1^, compared to other reported photocatalysts ranging from 1.6 to 21.3 mg (mg·kW·h)^−1^ (Table S2, Supporting Information).^[^ ^19^ ^]^ Collectively, these findings underscore the exceptional potential of g‐C_3_N_4_/CD‐10 as a highly effective photocatalyst for LEV degradation in water.

The contributions of various reactive species to the photocatalytic degradation of LEV were systematically elucidated through a series of radical scavenging experiments. As shown in Figure 2b, the removal of LEV decreased to 93%, 87%, 48%, and 53% upon the addition of •OH, h^+^, •O_2_^−^, and ^1^O_2_ quenchers using isopropanol (IPA), sodium oxalate (SA), p‐benzoquinone (p‐BQ), and furfuryl alcohol (FA), respectively. The corresponding rate constants are displayed in Figure S6b (Supporting Information). Quantitative analysis revealed the relative contributions of •OH, h^+^, •O_2_^−^ and ^1^O_2_ to be 6.2%, 17.0%, 42.0%, and 34.8%, respectively. The presence of predominant reactive species was further corroborated by the ESR spectra (Figure 2c–e), which detected the generation of h^+^, •O_2_^−^, and ^1^O_2_ in the g‐C_3_N_4_/CD‐10 system under visible light irradiation but showed a scarcity of •OH signals (Figure 2f). Given that the oxidation potential of h^+^ (*E*_VB_ = 2.11 V vs NHE) was lower than the *E*_0_ (•OH/H_2_O) (2.68 V vs NHE), the generation of •OH from water oxidation by h^+^ was thermodynamically unfavorable.^[^ ^20^ ^]^

**Figure 3:**
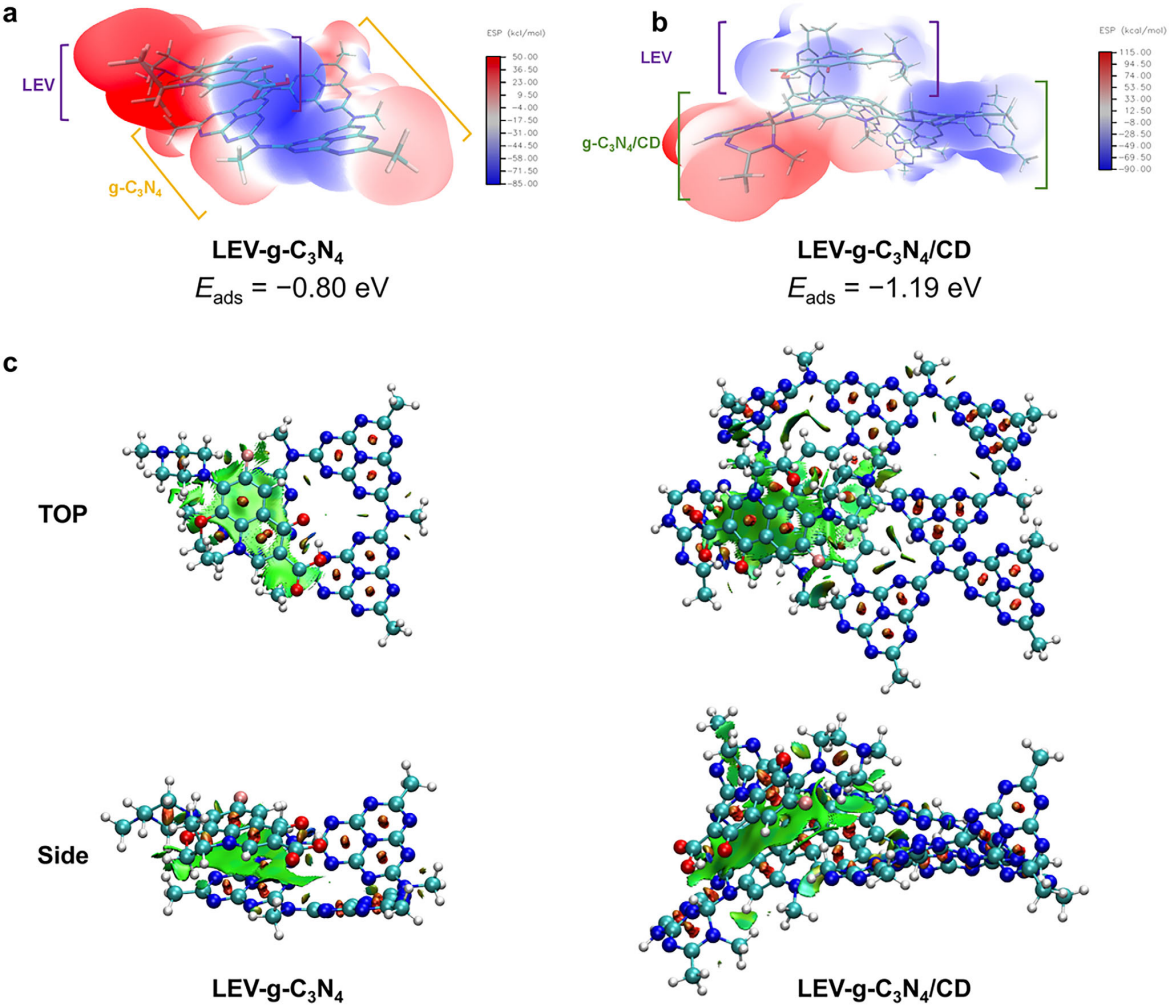
Electrostatic potential (ESP) maps and adsorption energies of a) LEV‐g‐C_3_N_4_ and b) LEV‐g‐C_3_N_4_/CD. c) 3D NCI surface plots of LEV‐CN and LEV‐g‐C_3_N_4_/CD (The green surface represents the NCI between LEV and the adsorbent).

The incorporation of CD onto g‐C_3_N_4_ increased the sorption strength between LEV and the photocatalyst. In the present study, the optimized geometries of the photocatalysts and the adsorption energies (*E*_ads_) revealed that the *E*_ads_ of LEV for g‐C_3_N_4_/CD‐10 (−1.19 eV) was stronger than for g‐C_3_N_4_ (−0.80 eV) (**Figures** **3**a,b). The enhanced adsorption strength can be partially attributed to the stronger noncovalent π–π interactions in LEV‐g‐C_3_N_4_/CD as revealed by a noncovalent interaction (NCI) plot analysis (**Figure** **3**c), which is partially due to the presence the aromatic π‐surface of CD. In addition, the presence of hydroxyl and amino groups on the CD surface provided hydrophilic functionality to g‐C_3_N_4_/CD, as evidenced by the FT‐IR spectra and the decreased contact angle after CD incorporation (Figure S7a; Figure S8, Supporting Information), which induce stronger dipole‐dipole and C─H•••π interactions that contribute to the increased adsorption strength of LEV onto g‐C_3_N_4_/CD. The enhanced adsorption strength between LEV and g‐C_3_N_4_/CD‐10 would facilitate the contact between LEV and reactive species such as h^+^, thereby beneficial for the photodegradation of LEV.

On top of the photocatalytic results, the proposed mechanism for the photocatalytic degradation of LEV by g‐C_3_N_4_/CD‐10 involved the following key steps. Upon photoexcitation, g‐C_3_N_4_/CD‐10 generated electron‐hole pairs (Equation 1). Since the CBM potential of g‐C_3_N_4_/CD‐10 was more negative than that of the O_2_/•O_2_^−^ redox couple (−0.52 eV vs −0.33 eV), it enabled the reduction of O_2_ to •O_2_^−^ (Equation 2).^[^ ^21^ ^]^ Furthermore, the •O_2_^−^ can then react with the h^+^ to form ^1^O_2_ (Equation 3), which along with the h^+^ themselves, can directly oxidize LEV (Equation 4).

(1) $$g-C_{3}N_{4}/\mathrm{CD}-10+\mathrm{hv}\to h^{+}+e^{-}$$

(2) $$e^{-}+O_{2}\to \cdot O_{2^{-}}$$

(3)

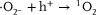



(4)

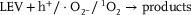




**Figure 4:**
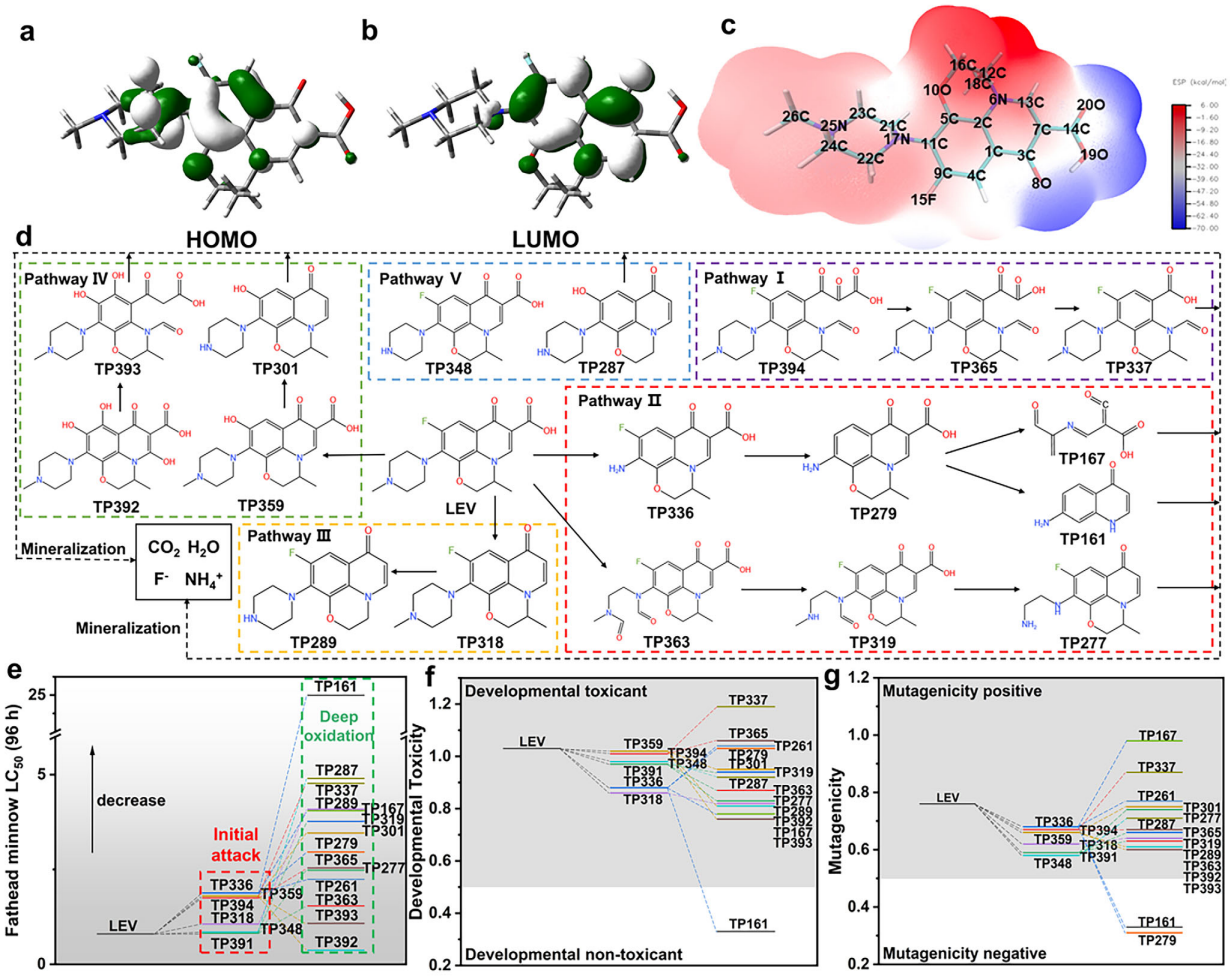
a) Spatial plots (isovalue = 0.03) of the highest occupied molecular orbital (HOMO) and the b) lowest unoccupied molecular orbital (LUMO) of LEV optimized at the B3LYP/6‐31+G(d,p) level. c) Electrostatic potential (ESP) surface of LEV. d) Possible pathways for LEV degradation by g‐C_3_N_4_/CD‐10. The toxicity of LEV and its degradation products include e) fathead minnow LC_50_ (96 h), f) developmental toxicity, and g) mutagenicity.

To elucidate the degradation pathway of LEV under LED light illumination, the transformation/degradation products were analyzed by liquid chromatograph‐mass spectrometry (LC‐MS). Together with the HOMO‐LUMO distribution and surface electrostatic potential (ESP) mapping of LEV (**Figure** **4**a−c), the Fukui index was calculated to further determine the active sites (Table S3 and Figure S9, Supporting Information). The ESP of LEV showed that the electron‐rich region (blue region) was located in the carboxylic group while the electron‐deficient region (red region) on the methyl‐1,4‐oxazine group, respectively. Particularly, the HOMO was localized on the π orbital of the benzene ring mixing with the nonbonding orbital of the piperazine N, while the LUMO was on the π* orbital of the 2,3‐dihydro‐7H‐[1,4]oxazino[2,3,4‐ij]quinoline‐7‐one moiety, indicating these regions susceptible to electrophilic and nucleophilic attacks, respectively.

The Fukui index indicated that atoms C1, C4, C5, O10, C11, N17, H38, and H41 were vulnerable to electrophilic attacks with relatively high f^−^ values, while atoms C3, C4, C5, O8, C11, C13, and N17 were susceptible to radical attacks with relatively high f^0^ values. Based on the theoretical calculations and the intermediate products identified by LC‐MC analysis, five degradation pathways of LEV oxidation were proposed involving the cleavage of the piperazine, benzene, and quinolone rings (Table S4 and Figure 4d, Supporting Information). Specifically, the N17 atom with high Fukui index (f^−^ = 0.1543, f^0^ = 0.0858) represents the most active site for electrophilic and radical attacks (e.g., h^+^ and •O_2_^−^), indicating that pathway II (dealkylation, product m/z  =  336.1369) and pathway V (demethylation, product m/z  =  348.1371) should be the primary pathways for LEV degradation. In addition, the C13 atom with a f^0^ value of 0.0464 might also be directly oxidized by •O_2_^−^, leading to the transformation of the quinolone ring in pathway I (product m/z  =  394.3311). Alternatively, the attack of ^1^O_2_ might result in the delocalization of the adjacent carboxylic acid group, as depicted in pathway IV (m/z = 318.1627). The TOC result showed that over 20% LEV was mineralized within 120 min under LED visible‐light irradiation by g‐C_3_N_4_/CD‐10, where the partially oxidized small molecules were further examined for their ecological risks.

The Toxicity Estimation Software Tool was utilized to assess the quantitative structure‐activity relationships and evaluate the toxicity of LEV and its degradation intermediates, specifically focusing on fathead minnow LC_50_ (96 h), developmental toxicity, and mutagenicity.^[^ ^22^ ^]^ As depicted in Figure 4e, LEV is classified as highly toxic with an LC_50_ (96 h) value of 0.8 mg L^−1^. Notably, the primary degradation products exhibited lower toxicity than LEV, and further oxidation resulted in low‐toxicity compounds, such as TP161 with an LC_50_ value of 25 mg L^−1^. Developmental toxicity assessments (Figure 4f) indicated that LEV is toxic (value of 1.03), while the initial degradation products showed reduced but still notable toxicity, ultimately becoming non‐toxic after deep oxidation, as exemplified by TP161. Regarding mutagenicity (Figure 4g), most degradation products were attenuated or detoxified to values below the 0.5 threshold, indicating a reduction in mutagenic potential compared to LEV. These findings confirm that photocatalytic degradation of LEV by g‐C_3_N_4_/CD‐10 could effectively mitigate ecological risks.

**Figure 5:**
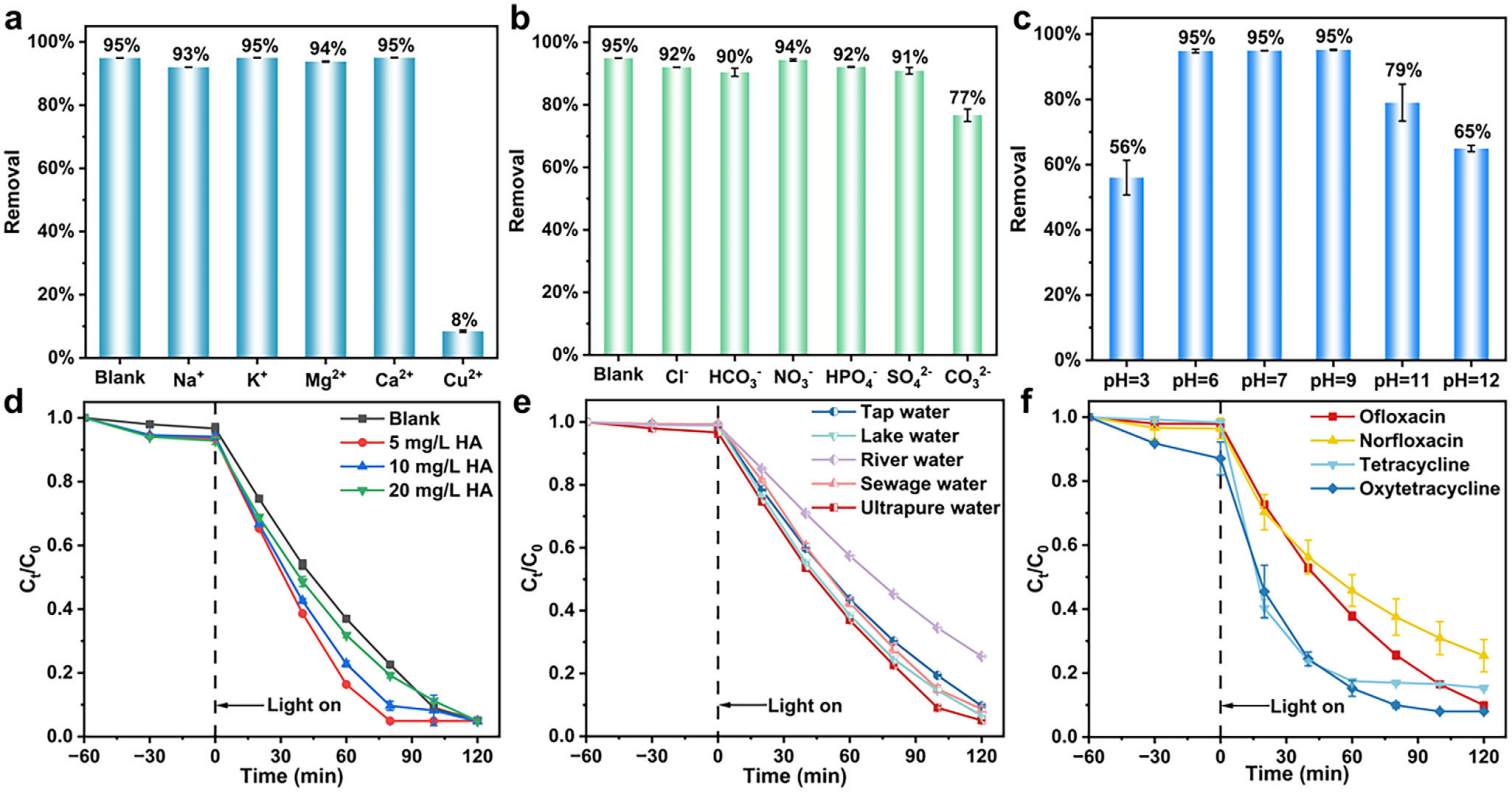
The 10 W LED‐driven photocatalytic degradation of LEV under g‐C_3_N_4_/CD‐10 photocatalyst, with the influence of a) cation, b) anion species (ionic concentration = 10 mM), c) solution pH values, d) humic acid, and e) various water sample. f) Removal of different antibiotics by g‐C_3_N_4_/CD‐10.

The influence of various water chemistry factors on LEV removal, including inorganic ions, natural organic matter, and pH was comprehensively studied to simulate real wastewater and natural aquatic environments. As shown in **Figure** **5**a, Na^+^, K^+^, Mg^2+^, and Ca^2+^ showed no apparent impact on LEV removal. In contrast, the presence of transition metal Cu^2+^ substantially decreased the removal efficiency of LEV, likely by scavenging CBM electrons required for •O_2_^−^ formation and thereby hampering the subsequent photocatalytic process.^[^ ^23^ ^]^ The existence of inorganic anions such as Cl^−^, SO_4_^2−^, HCO_3_^−^, NO_3_^−^, and HPO_4_^−^ barely affected LEV removal efficiency, while the presence of CO_3_^2−^ significantly reduced the LEV removal rate to 77% (Figure 5b). The inhibitory effect observed with CO_3_^2−^ can be primarily attributed to the change of solution pH (from 7.2 to 11.0), which aligned closely with the photocatalytic tests conducted under varying pH conditions (Figure 5c). Specifically, the removal of LEV by g‐C_3_N_4_/CD‐10 remained steady across a broad pH range from 6.0 to 9.0 but decreased to 55%, 79%, and 65% at pH = 3.0, 11.0, and 12.0, respectively. This observation could be partially attributed to the dynamics of electrostatic interaction between LEV and the photocatalyst under various pH environments. The LEV molecule exhibits two dissociation constant values (p*K*a_1_ = 6.0, p*K*a_2_ = 8.2),^[^ ^24^ ^]^ while the isoelectric point of g‐C_3_N_4_/CD‐10 was measured as 4.2 (Figure S6c, Supporting Information), resulting in stronger electrostatic repulsion at pH = 3 or 12 and hindering LEV removal. Additionally, strong acidic or alkaline environments could quench reactive species, resulting in inferior photodegradation performance.^[^ ^25^ ^]^ Specifically, H^+^ would react with •O_2_^−^ to form H_2_O_2_ (•O_2_^−^ + H^+^ → HOO•, 2HOO• → H_2_O_2_ + O_2_) under acidic conditions, while excess OH^−^ would consume h^+^ (h^+^ + 4OH^−^ → O_2_ + 2H_2_O) under alkaline conditions, both leading to decreased LEV removal.

The effect of humic acid (HA), a major component of natural organic matter in the aquatic system, was investigated on the removal of LEV. Interestingly, the LEV removal rate was promoted in the addition of HA up to 20 mg L^−1^, which started to inhibit the removal afterward (Figure 5d; Figure S6d, Supporting Information). The promoting effect of HA could be attributed to the presence of multiple luminescent groups in the molecular structure of HA, such as benzenes, carbonyls, and carboxyl groups, which could be photoexcited to generate various reactive species contributing to LEV photodegradation.^[^ ^26^ ^]^ The EPR results confirmed the increased production of •OH upon the addition of HA (Figure S6e, Supporting Information), corroborating the beneficial effect of HA at moderate concentrations. However, further increasing the HA concentration caused a detrimental effect, which was likely due to the shading effect that reduced light absorption by the photocatalyst (Figure S6f, Supporting Information).

Various real water samples (e.g., tap water, sewage water, lake water, and river water sourced locally) were tested to verify the photocatalytic performance of g‐C_3_N_4_/CD‐10 under environmentally relevant conditions. The characteristics of the water samples are detailed in Table S5 (Supporting Information). Despite the presence of inherent dissolved organic matter and/or associated ions in real water matrices, g‐C_3_N_4_/CD‐10 demonstrated excellent photocatalytic capability, achieving over 75% LEV removal within 120 min in all four real water sources (Figure 5e). Furthermore, g‐C_3_N_4_/CD‐10 also presented broad applicability to other antibiotics, for instance, ofloxacin (OFL) and oxytetracycline (OTC) removal were over 90%, followed by tetracycline (TC) of 85%, and norfloxacin (NOR) of 75% (Figure 5f). The photocatalyst g‐C_3_N_4_/CD‐10 remained high LEV removal efficiency above 90% over four consecutive reaction cycles (Figure S10, Supporting Information), where the structure and function stability were further confirmed by FT‐IR, XRD and UV–vis spectra (Figure S11, Supporting Information). These results clearly indicate the versatility, applicability, and stability of g‐C_3_N_4_/CD‐10 photocatalyst for practical application to real‐world water samples.

**Figure 6:**
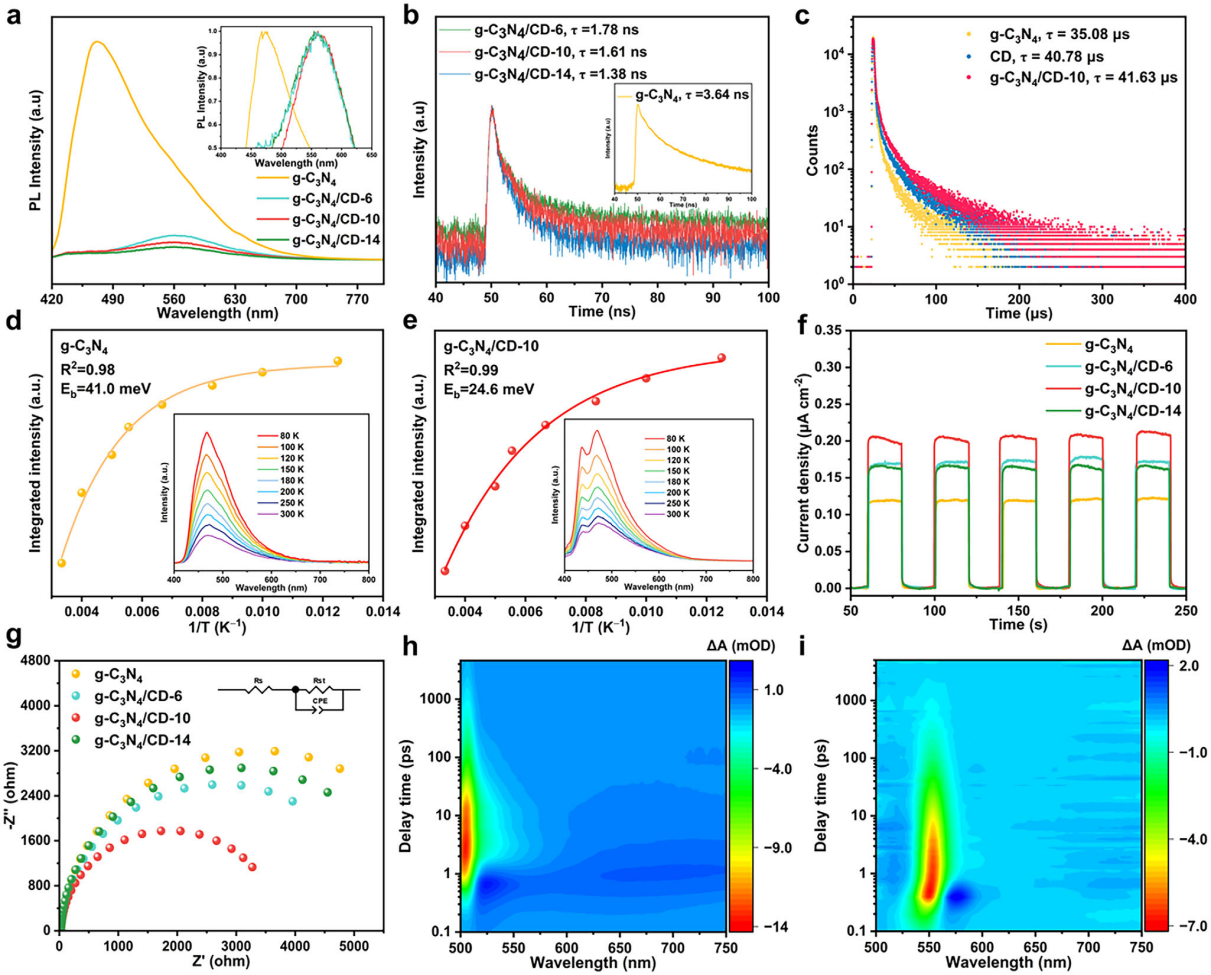
a) PL spectra (insert: normalized PL spectra) and b) time‐resolved transient fluorescence spectrometry (TRPL) of g‐C_3_N_4_, g‐C_3_N_4_/CD‐6, g‐C_3_N_4_/CD‐10, and g‐C_3_N_4_/CD‐14. c) Delayed emission lifetime decays of g‐C_3_N_4_, CD, and g‐C_3_N_4_/CD‐10. Integrated PL intensity as a function of temperature (insert: temperature‐dependent PL spectra from 80 to 300 K) of d) g‐C_3_N_4_, and e) g‐C_3_N_4_/CD‐10. f) Transient photocurrent responses and g) EIS Nyquist patterns of g‐C_3_N_4_, g‐C_3_N_4_/CD‐6, g‐C_3_N_4_/CD‐10, and g‐C_3_N_4_/CD‐14. TAS measurements for h) g‐C_3_N_4_ and i) g‐C_3_N_4_/CD‐10 under 365 nm excitation.

The photophysical and photoelectrochemical properties of the g‐C_3_N_4_ and g‐C_3_N_4_/CD materials were thoroughly investigated to gain deeper insights into their role in the observed photodegradation performance. Upon photoexcitation, the pristine g‐C_3_N_4_ sample exhibited a structureless emission band peaking at 470 nm (**Figure** **6**a). Doping CD into g‐C_3_N_4_ red‐shifted the emission band by 30 nm, independent of the CD dopant concentrations (6−14%). The bathochromic shift is primarily attributed to the transition from the LE state of g‐C_3_N_4_ to the predominant CT excited state of g‐C_3_N_4_/CD, as supported by our computational results and related studies.^[^ ^27^ ^]^ Compared to pristine g‐C_3_N_4_, the g‐C_3_N_4_/CD displayed drastically reduced photoluminescence (PL) intensity, possibly governed by the energy gap law.^[^ ^28^ ^]^ When CD doping concentration increased from 6 to 14%, the PL intensity further decreased, suggesting suppressed recombination of photogenerated electron‐hole pairs via the minor perturbation of the excited characters due to the increasing of the CT character.

Transient PL spectra of all the samples revealed a short component in the nanosecond regime (Figure 6b) and a long component in the microsecond regime as displayed in the corresponding time‐delayed emission spectra (Figure 6c). For the nanosecond process, the average lifetimes of photogenerated charge were determined to be 3.64 ns for pristine g‐C_3_N_4_ and ≈1.38−1.78 ns for the g‐C_3_N_4_/CD samples (Figure 6b). The shorter lifetimes observed for the g‐C_3_N_4_/CD samples indicated the successful manipulation of the excited state character via CD doping.^[^ ^29^ ^]^ As shown in Figure 6c, the delayed emission component for g‐C_3_N_4_, CD, and g‐C_3_N_4_/CD‐10 fell within the range of 35.0 to 41.6 µs. Drawing from relevant literature and structurally related studies on g‐C_3_N_4_ materials, the possible participation of a triplet excited state, potentially activated via an intersystem crossing process, cannot be excluded in the present investigations.^[^ ^30^ ^]^ This prolonged excited state lifetime would only promise to enhance photocatalytic efficiency by facilitating a greater proportion of excitons to dissociate into free charge carriers,^[^ ^31^ ^]^ underscoring the importance of increased CT excited state character in the system.

The exciton binding energy (*E*_b_), a fundamental parameter capturing the Coulombic interaction of excitons, was determined from the temperature‐dependent PL spectra (Figures 6d&e and Figure S12, Supporting Information).^[^ ^32^ ^]^ The obtained *E*_b_ values of g‐C_3_N_4_/CD samples (19.1−25.7 meV) were much smaller than that of g‐C_3_N_4_ (41.0 meV), suggesting easier dissociation of excitons into free electron‐hole pairs. Furthermore, photoelectrochemical measurements were conducted to further elucidate the charge separation and mobility. All the g‐C_3_N_4_/CD samples showed stronger photocurrent responses than the g‐C_3_N_4_ (Figure 6f), consistent with the decreased *E*_b_ values upon CD doping that increased charge carrier mobility. As evident from the diminished arc radius in the electrochemical impedance spectra (Figure 6g), CD decorated onto the g‐C_3_N_4_ reduced the charge transfer resistance, which is potentially beneficial to improve the charge separation efficiency of photoinduced carriers. However, when the CD doping level exceeds a certain level (10 wt.%), more pronounced defects and disruption of the interlayer ordering in g‐C_3_N_4_, as previously discussed (Figure 1d), would negatively impact carrier recombination, leading to larger charge transfer resistance and a reduced photocurrent response in g‐C_3_N_4_/CD‐14.^[^ ^33^ ^]^

The photoinduced catalytic process generally initiated with an ultrafast charge separation process occurred on the picosecond (ps) to nanosecond (ns) time scales.^[^ ^34^ ^]^ Time‐resolved transient absorption spectroscopy (TAS) was employed to monitor the ultrafast dynamic processes of photogenerated charge carriers in g‐C_3_N_4_ and g‐C_3_N_4_/CD‐10. The TAS measurements revealed a negative ΔA signal at 500 nm and a positive ΔA feature at wavelengths beyond 525 nm for pristine g‐C_3_N_4_ with a time constant of ≈τ1 = 34.5 ps and τ2 = 229.7 ps (Figure 6h and Figure S13, Supporting Information), whereas g‐C_3_N_4_/CD‐10 displayed red‐shifted bands with a negative ΔA signal at 560 nm and two positive ΔA bands peaking at 525 nm and 580 nm (Figure 6i and Figure S13, Supporting Information). As indicated by the steady state ground state absorption spectra (Figure 1e), the absorption peaks for both g‐C_3_N_4_ and g‐C_3_N_4_/CD‐10 systems are below 500 nm, suggesting that the primary ground state bleaching signals for both systems likely fall beyond the detection range of our instrument. However, we found a good match between the steady state emission signals and the negative ΔA signals for both systems, which we tentatively assign to stimulated emission signals arising from the emissive excited states. The positive ΔA signals are attributed to photoinduced excited state absorption features. The significant red‐shift of both stimulated emission and excited state absorption bands of g‐C_3_N_4_/CD‐10 is consistent with our assignment that there is population of a CT excited state, which has lower energy than the LE excited state in g‐C_3_N_4_. These distinctive transient absorption characteristics of g‐C_3_N_4_ and g‐C_3_N_4_/CD‐10, together with the DFT/TD‐DFT calculations discussed in the previous section, support the tentative assignment of the co‐existing LE and CT excited states in g‐C_3_N_4_/CD‐10 and their stimulated emission.

Overall, the incorporation of CD into g‐C_3_N_4_ altered the excited state characteristics of the photocatalysts, leading to a slight shortening of the excited state dynamics (from 3.64 to 1.38 ns) as observed in the TAS and TRPL measurements in this study. Importantly, despite such reduction, the excited state lifetimes in the picosecond to nanosecond range remained adequate to facilitate interfacial charge transfer processes from the edge carriers.^[^ ^35^ ^]^ This finding underscored that the slightly reduced lifetime did not adversely impact photocatalytic performance; rather it highlighted the critical role of CT excited state in enhancing photocatalytic activity. In view of the inherent competition between exciton formation and charge carrier generation in photocatalysis, the shift from LE to CT state in the g‐C_3_N_4_/CD‐10 system, arising from asymmetric electron density distributions between π‐conjugated domains of CD and tri‐s‐triazine units of g‐C_3_N_4_, weakens Coulombic interactions in the photocatalyst system. Consequently, the involvement of CT state reduces *E*_b_ and facilitates exciton dissociation into free charge carriers and improving charge separation efficiency as sustained by photoelectrochemical measurements, a crucial factor for enhancing photocatalytic performance.

## 3 Conclusion

This study showcases that CD functionalized g‐C_3_N_4_ can regulate exciton dissociation by perturbing a CT excited state in the system, confirmed by DFT and TD‐DFT calculations and temperature‐dependent photoluminescence spectra. Specifically, g‐C_3_N_4_/CD‐10 exhibits a 3−fold increase in the degradation rate of LEV under 10 W LED irradiation, achieving over 95% removal within 120 min, outperforming pristine g‐C_3_N_4_. This enhancement is attributed to the enhanced CT excited state character and a suitable band structure, which modulates the exciton binding energy and facilitating charge transfer. Furthermore, g‐C_3_N_4_/CD‐10 exhibited reliable performance across various water samples from the actual environment and retained its structural integrity over multiple cycles. This work not only advances the understanding of exciton dynamics within g‐C_3_N_4_‐based materials but also offers valuable insights through the lens of the manipulation of the charge transfer excited state for improved photocatalytic activity for the g‐C_3_N_4_‐based system.

## Conflict of Interest

The authors declare no conflict of interest.

## Supporting information



Supporting Information

## Data Availability

The data that support the findings of this study are available from the corresponding author upon reasonable request.
