# Supplementary material for: Manipulating Charge Dynamics in Carbon Nitride by Carbon Dot Doping for Efficient Photocatalysis
Source: Adv Sci (Weinh). 2025 Apr 26;12(25):2417390. doi: 10.1002/advs.202417390 (PMC12224990; doi:10.1002/advs.202417390)
Supplement: Supplementary file 1 — Supporting Information [file ADVS-12-2417390-s001.pdf]

ADVANCED SCIENCE
Open Access

Supporting Information

for *Adv. Sci.*, DOI 10.1002/advs.202417390

Manipulating Charge Dynamics in Carbon Nitride by Carbon Dot Doping for Efficient Photocatalysis

Lingfeng Ouyang, Maggie Ng, Zhang-Hong Zhou, Hao Wu, Man-Chung Tang and Season Si Chen**

Supporting Information

for

Manipulating Charge Dynamics in Carbon Nitride by Carbon Dot Doping for Efficient Photocatalysis

Lingfeng Ouyang[1], Maggie Ng[1], Zhang-Hong Zhou[2], Hao Wu[2], Man-Chung Tang[3,*], Season Si Chen[1,*]

[1] Institute of Environment and Ecology, Tsinghua Shenzhen International Graduate School, Tsinghua University, Shenzhen 518005, PR China.

[2] Macau Institute of Materials Science and Engineering (MIMSE), Faculty of Innovation Engineering, Macau University of Science and Technology, Macau, Taipa, 999078, PR China

[3] Institute of Materials Research, Tsinghua Shenzhen International Graduate School, Tsinghua University, Shenzhen 518005, PR China.

* Corresponding Author:

Dr. Kobe Man-Chung Tang (kobetang2021@sz.tsinghua.edu.cn)

Dr. Season Si Chen (season.chen@sz.tsinghua.edu.cn)

Number of Pages: 48

Number of Texts: 7

Number of Tables: 12

Number of Figures: 14

Table of Contents

Index	Caption	Page
**Text S1**	Experimental section	S5
**Text S2**	Characterization of photocatalysts	S8
**Text S3**	Electrochemical measurements	S9
**Text S4**	Details of reactive species detection	S10
**Text S5**	Transient absorption spectra (TAS) test	S12
**Text S6**	Degradation intermediates identification of LEV	S13
**Text S7**	Details of theoretical calculations	S14

Index	Caption	Page
**Table S1**	Computed S_r, D, and t indices in the electron excitation analysis of g-C_3N_4 and g-C_3N_4/CD at the optimized S_1 geometries	S16
**Table S2**	Efficiency comparison of g-C_3N_4/CD-10 and other reported photocatalysts for LEV degradation	S17
**Table S3**	Hirshfeld charges and Fukui index of LEV computed at the M06/6-31G(d,p) level	S19
**Table S4**	The information of degradation compounds of LEV	S21
**Table S5**	Characteristics of various water samples	S23
**Table S6**	LC settings for the determination of LEV intermediates	S24
**Table S7**	HPLC settings of different antibiotics	S24
**Table S8**	Cartesian coordinates of the geometry of LEV optimized at the M06/6-31G(d,p) level	S25
**Table S9**	Cartesian coordinates of the geometry of g-C_3N_4 optimized at the M06/6-31G(d,p) level	S26
**Table S10**	Cartesian coordinates of the geometry of g-C_3N_4/CD optimized at the M06/6-31G(d,p) level	S27
**Table S11**	Cartesian coordinates of the geometry of LEV-g-C_3N_4/CD optimized at the M06/6-31G(d,p) level	S29
**Table S12**	Cartesian coordinates of the geometry of LEV-g-C_3N_4 optimized at the M06/6-31G(d,p) level	S32

Index	Caption	Page
**Figure S1**	The optimized geometries of CD	S34
**Figure S2**	Electrostatic potential surface of (a) g-C_3N_4 and (b) g-C_3N_4/CD	S34
**Figure S3**	(a) TEM image of CD, (b) g-C_3N_4, and (c) g-C_3N_4/CD-10. (d) FT-IR spectra, (e) XPS survey spectra, and high-resolution XPS (f) C 1s, (g)N 1s, and (h) O 1s spectra of g-C_3N_4, g-C_3N_4/CD-6, g-C_3N_4/CD-10, and g-C_3N_4/CD-14	S35
**Figure S4**	Photographs of the CD under sunlight and 365 nm UV irradiation, g-C_3N_4, g-C_3N_4/CD-6, g-C_3N_4/CD-10, and g-C_3N_4/CD-14	S39
**Figure S5**	(a) Tauc plots, and (b) VB XPS plots of g-C_3N_4, g-C_3N_4/CD-6, g-C_3N_4/CD-10, and g-C_3N_4/CD-14	S39
**Figure S6**	(a) First-order constants of different photocatalyst. (b) The rate constants of LEV degradation curves on g-C_3N_4/CD-10 with different scavengers under equivalent reaction conditions. (c) Zeta potential of g-C_3N_4/CD-10 at different pH values. (d) The corresponding rate constants of LEV degradation curves over g-C_3N_4/CD-10 alone and with different HA concentrations. (e) ESR signals for •OH of g-C_3N_4/CD-10 under 5 mg/L HA conditions. (f) UV-vis absorption spectra of LEV and HA	S40
**Figure S7**	(a) FT-IR spectra, (b) XPS survey spectra, (c) high-resolution XPS C 1s spectra, (d) high-resolution XPS N 1s spectra, and (e) high-resolution XPS O 1s spectra of CD; (f) UV-vis absorption spectra (g) Excitation-emission PL spectra, and (h) up-conversion PL emission spectra of CD in aqueous solutions	S41
**Figure S8**	Contact angle of (a) g-C_3N_4 and (b) g-C_3N_4/CD-10	S43

**Figure S9**	Labeling of LEV	S43
**Figure S10**	Recycling degradation of LEV over g-C_3N_4/CD-10	S43
**Figure S11**	(a) FT-IR spectra, (b) XRD pattern spectra, and (c) UV–vis DRS spectra of g-C_3N_4/CD-10 before and after reaction	S44
**Figure S12**	Integrated PL intensity as a function of temperature (insert: temperature-dependent PL spectra from 80 to 300 K) of (a) g-C_3N_4/CD-6, and (b) g-C_3N_4/CD-14	S44
**Figure S13**	TAS measurements for (a) g-C_3N_4 and (b) g-C_3N_4/CD-10 under 365 nm excitation, and corresponding decay curves of normalized TAS of (c) g-C_3N_4 and (d) g-C_3N_4/CD-10 at 550 nm.	S45
**Figure S14**	(a) SEM image of g-C_3N_4 and SEM/EDS mapping of C, N, and O elements in g-C_3N_4. (b) SEM image of g-C_3N_4/CD-10 and SEM/EDS mapping of C, N, and O elements in g-C_3N_4/CD-10	S46

Text S1. Experimental section

Chemicals and reagents

Levofloxacin (LEV), ofloxacin (OFL), ciprofloxacin (CIP), norfloxacin (NOR), tetracycline (TC), oxytetracycline (OTC), and humic acid (HA, fulvic acid > 90%) were purchased from Macklin Reagent (Shanghai, China). Methanol and formic acid were provided by Sigma-Aldrich (St. Louis, MO, USA). Melamine, ammonium citrate (AC), ethylenediamine, isopropanol (IPA), p-benzoquinone (p-BQ), furfuryl alcohol (FA), and sodium oxalate ($Na_2C_2O_4$, SA) were obtained from Aladdin Chemical (Shanghai, China). All the chemical reagents were used as received without further purification. The ultrapure water used in this work was obtained from a Milli-Q® Integral system (18.2 MΩ·cm, 25°C).

Synthesis of photocatalysts

The carbon dots (CD) were prepared via a modified hydrothermal based on previous studies.[1] Specifically, 10 mmol of ammonium citrate was dissolved in 20 mL of ultrapure water, followed by the addition of 670 µL of ethylenediamine and stirred thoroughly for 30 min. The solution was then transferred to a Teflon-lined autoclave and remained at a 200 °C oven for 5 h. The dark brown solution was filtered through a 0.22-µm nylon membrane after cooling down. To ensure the purity and uniformity of the CD, the filtrate underwent 24 h of dialysis (MWCO 500) and subsequent freeze-drying to obtain both the pure solution (*c.a.* 85 mg mL^{-1}) and the solid form of CD.

The CD-modified carbon nitride (g-C_3N_4/CD) was synthesized via a facile polymerization method. Typically, different volumes of the CD pure solution were

diluted to 3 mL with ultrapure water, and the diluted solution was subsequently mixed thoroughly with 20 mmol of melamine powder. The mixture was transferred to a 20 mL crucible and dried overnight at 80 °C to form a solid precursor. The brown solid obtained was heated to 550 °C at a heating rate of 3 °C min^{-1} and then kept for 4 h under air atmosphere in a muffle furnace (Hefei Kejing, KSL-1100X). After cooling to room temperature, the "g-C_3N_4/CD-x" product was ground into powder for further use, where "x" represents the mass doping percentages of CD relative to the yield of g-C_3N_4, which is approximately 1.21 g from 20 mmol melamine. Graphitic carbon nitride was also synthesized using the same method without CD. To verify the scalability and reproducibility of this synthesis method, photocatalytic experiments were performed using several independent batches of synthesized g-C_3N_4 and g-C_3N_4/CD.

Photocatalytic degradation of antibiotics

Batch photocatalytic experiments of antibiotics degradation were conducted using a multi-channel photochemical reaction system (PCX-50C, Perfect Light, Beijing, China) equipped with a 10 W white LED lamp (400–800 nm, 450 mW cm^{-2}), with the reaction temperature maintained at 25 °C via a circulating water bath. Levofloxacin served as the target pollutant in this study, with its photocatalytic degradation performance evaluated. Typically, 25 mg of photocatalyst was dispersed in 50 mL of 10 mg L^{-1} LEV solution and stirred at 400 rpm for 1 h under dark conditions to reach adsorption-desorption equilibrium. At designated time intervals, 1 mL of supernatant was rapidly extracted from the reactor and filtered through a 0.22-μm nylon membrane. The photocatalytic degradation reaction conditions for various antibiotic solutions were

kept consistent with the one for LEV. The concentrations of all antibiotic pollutants were quantified using high-performance liquid chromatography (HPLC, Shimadzu LC-2050C) with a ShimNex CS C18 column (5 μm, 4.6 × 250 mm) and a PDA detector, as detailed in Supplementary Table S1. The mineralization of LEV was evaluated using a total organic carbon analyzer (TOC, Shimadzu TOC-L). Photocatalyst reusability was assessed over four consecutive cycles, wherein the photocatalysts were recovered after each cycle by filtration through a 0.22-μm nylon membrane, rinsed with ethanol and ultrapure water, and dried overnight at 60 °C prior to the next photocatalytic run. All the photocatalytic experiments were conducted in triplicate.

Text S2. Characterization of photocatalysts

The crystal structure of g-C_3N_4 and g-C_3N_4/CD samples was characterized by using X-ray diffraction (XRD, Rigaku Ultima IV) with nickel-filtered Cu Kα radiation in the range of 5−80 ° with a scanning rate of 2 ° min^{-1}. Fourier transform infrared (FT-IR) spectra of CD and g-C_3N_4/CD were recorded using the JASCO FT/IR-4100 spectrophotometer. Raman spectra of CD were obtained using the WITec alpha300R at room temperature with a 785 nm solid-state laser as the excitation source. The morphology and microstructure of all samples were characterized using a scanning electron microscope (SEM, HITACHI SU8010) and FEI spirit T12 transmission electron microscopy (TEM). X-ray photoelectron spectra (XPS) were acquired on the Thermo Scientific K-Alpha, and the binding energy of C 1s (284.4 eV) was used as a calibration standard. The ultraviolet−visible (UV-vis) absorption spectra were measured by a UV spectrophotometer (Shimadzu UV-2600). The photoluminescence (PL) properties of the CD solution were tested by a fluorescence spectrophotometer (Hitachi F-4700). A steady-state fluorescence spectrometer (Edinburgh FLS1000) was used to test the fluorescence quantum yield of CD powders, the temperature-dependent PL spectra, and delayed emission spectra of photocatalysts. For PL spectra and transient fluorescence lifetime characterization of g-C_3N_4 and g-C_3N_4/CD, the OmniFluo990HEL transient fluorescence spectroscopy was employed with an excitation wavelength of 375 nm. The UV-vis diffuse reflectance (UV-DRS) spectra of photocatalyst samples were recorded using the PerkinElmer Lambda950 spectrophotometer.

Text S3. Electrochemical measurements

The electrochemical spectra were measured on the electro-chemical workstation (CHI760E) in the 0.2 M Na_2SO_4 aqueous solution as the electrode, where the cleaned indium tin oxide (ITO) deposited with photocatalyst samples, a Pt flake, and a saturated calomel electrode were adopted as the working electrodes, counter electrode, and reference electrode, respectively. Electrochemical impedance spectroscopy (EIS) measurements were taken over a frequency range of 0.01 Hz to 100 kHz with a sinusoidal potential amplitude of 5 mV per application.

Text S4. Details of reactive species detection

To detect the reactive species (RS) generated during the photocatalytic reaction, different compounds including isopropanol (IPA), p-benzoquinone (p-BQ), furfuryl alcohol (FA), and sodium oxalate (SA) were introduced into the LEV solution as scavengers of hydroxyl radicals (•OH), superoxide radicals ($\bullet O_2^-$), singlet oxygen ($^1O_2$), and photogenerated holes (h^+), respectively. The concentrations of the different scavengers were 5 mM, and other conditions were consistent with the photocatalytic experiments described above. The contributions of each RS to the overall LEV photodegradation kinetics were calculated using Equations (1–8):

$$\alpha_{\bullet OH} = \frac{k_{\bullet OH}}{k} \approx \frac{(k - k_{IPA})}{k} \quad (1)$$

$$\alpha_{\bullet O_2^-} = \frac{k_{\bullet O_2^-}}{k} \approx \frac{(k - k_{p-BQ})}{k} \quad (2)$$

$$\alpha_{\ ^1O_2} = \frac{k_{\ ^1O_2}}{k} \approx \frac{(k - k_{FA})}{k} \quad (3)$$

$$\alpha_{h^+} = \frac{k_{h^+}}{k} \approx \frac{(k - k_{SA})}{k} \quad (4)$$

$$R_{\bullet OH} = \frac{\alpha_{\bullet OH}}{\alpha_{\bullet OH} + \alpha_{\bullet O2^-} + \alpha_{\ ^1O_2} + \alpha_{h^+}} \quad (5)$$

$$R_{\bullet O_2^-} = \frac{\alpha_{\bullet O_2^-}}{\alpha_{\bullet OH} + \alpha_{\bullet O2^-} + \alpha_{\ ^1O_2} + \alpha_{h^+}} \quad (6)$$

$$R_{\ ^1O_2} = \frac{\alpha_{\ ^1O_2}}{\alpha_{\bullet OH} + \alpha_{\bullet O2^-} + \alpha_{\ ^1O_2} + \alpha_{h^+}} \quad (7)$$

$$R_{h^+} = \frac{\alpha_{h^+}}{\alpha_{\bullet OH} + \alpha_{\bullet O2^-} + \alpha_{\ ^1O_2} + \alpha_{h^+}} \quad (8)$$

where α_{RS} and R_{RS} are the inhibition rate and relative contribution of different RS to LEV degradation, respectively; k_{RS} is the first-order constant for LEV degradation when different RS scavengers were added.

The electron spin resonance (ESR) signals of RS spin-trapped were operated on the Bruker EMXplus-6/1 spectrometer. For the qualitative distinguished of the RS (•OH,

$\bullet O_2^-$, $^1O_2$ and h^+), the signals of •OH and $\bullet O_2^-$ generation in the photocatalytic system were monitored using 5,5-dimethyl-1-pyrroline-N-oxide (DMPO) as a spin trapping agent. Moreover, 2,2,6,6-Tetramethylpiperidine (TEMP) and 2,2,6,6-Tetramethylpiperidine 1-oxyl (TEMPO) were used to test the signal of $^1O_2$ and h^+, respectively.

Text S5. Transient absorption spectra (TAS) test

Powdered samples were dispersed in ethanol at a concentration of 0.5 mg ml^{-1}, and the suspensions were spun-coated onto a 2 × 2 cm quartz glass using a spin-coater and allowed to dry naturally at room temperature to obtain thin-film samples. All fs-TAS measurements were performed on a femtosecond pump-probe transient absorption (TA) spectrometer. The device consisted of a 1 KHz Ti:Sapphire laser amplification system (Spitfire Ace, Spectra Physics, Inc.), which outputs a beam with a center wavelength of 800 nm and a pulse width of 80 fs. The output beam was split into two. One part goes into the optical parametric amplification system (TOPAS Prime, Light Conversion), and the other part is focused to the CaF_2 crystal, which produces a continuous wave of white light with wavelengths ranging from 500 nm to 750 nm as the probe beam. During the measurements, the pump beam at 365 nm with energy of 200 nJ per pulse was combined with the probe beams in the thin-film sample. Signals were collected in the transmission geometry and TA measurements were performed with an overall time resolution of 100 fs. All experiments were performed at room temperature.

Text S6. Degradation intermediates identification of LEV

The photodegradation of LEV was described by a first-order kinetic model as shown in Equation (9):

$$\ln\left(\frac{C_t}{C_0}\right) = -kt \tag{9}$$

where C_0 and C_t (mg/L) are the initial and time t (min) LEV concentrations, respectively; k (min^{-1}) is the first-order constant for LEV degradation.

The intermediates of LEV degradation were analyzed by liquid chromatography-mass spectrometry (LC-MS, Agilent 1290, Agilent QTOF 6550) equipped with a Waters BEH C18 column (1.7 μm, 2.1 × 100 mm). The analysis method for LEV degradation intermediates was consistent with a previous study.[2] Specifically, the ion source pseudo-electrospray interface (ESI+) with fragment scanning mode of positive ion spray with a mass range of 20-500 m/z. The capillary voltage was set to 4 kv. The sheath gas temperature and flow rate were set to 350 °C and 12 L/min, respectively. The details are listed in Table S6.

Text S7. Details of theoretical calculations

Density functional theory (DFT) calculations were performed with the Gaussian 16 suite of programs.[3] The ground-state (S_0) geometry of LEV was fully optimized in water with the M06 functional,[4] which includes empirical dispersion corrections and is able to handle dispersion interactions effectively, and the 6-31G(d,p) basis set,[5] in conjunction with the solvation model density (SMD) continuum method.[6] The SMD model assumes that the solvent is a continuous and homogeneous medium with a known dielectric constant. It incorporates both electrostatic and non-electrostatic interactions between the solute and solvent, so it was used to model the solvation effects in water, where both electrostatic and dispersion forces significantly influence molecular structure and stability. Analysis of the Fukui indices and generation of the electrostatic potential (ESP) surface of LEV were performed by Multwfn.[7] To compute the adsorption energy, the geometries of CD, g-C_3N_4, g-C_3N_4/CD, and the associated complexes with LEV were optimized in water at the same level of theory. Vibrational frequency calculations were performed on the optimized geometries and all stationary points were verified to be minima on the potential energy surface, as there were no imaginary frequencies observed. The adsorption energy (E_{ads}) of the system is calculated by the following equation,

$$E_{ads} = E_{Total} - E_A - E_B \tag{10}$$

where E_{Total} is the energy of the complex and E_{A} and E_{B} are the energies of the separated molecules. To investigate the noncovalent interactions (NCI) between LEV and CN as well as NCCN, NCI analysis of the complexes has been performed based on

the geometries optimized at the M06/6-31G(d,p) level. To calculate the exciton binding energies and the amount of charge transfer (CT) in g-C_3N_4 and g-C_3N_4/CD, their S_0 and the lowest singlet excited (S_1) states were optimized in water by DFT and Time-dependent density functional theory (TD-DFT)[9] in the Tamm-Dancoff approximation (TDA),[10] respectively, with the long-range corrected B3LYP functional using the Coulomb-attenuating method (CAM-B3LYP),[11] which is widely used to study CT processes due to its accurate treatment of long-range electron-electron interactions for properly describing CT excited states. The exciton binding energies, CT percentages, as well as the S_r, D, and t indices were then calculated in the electron excitation analysis by Multiwfn.[12] The exciton binding energies of g-C_3N_4 and g-C_3N_4/CD were computed to be 4.11 and 3.47 eV, respectively. The Cartesian coordinates of the geometries of LEV, g-C_3N_4, g-C_3N_4/CD, the complex of LEV and g-C_3N_4, as well as the complex of LEV and g-C_3N_4/CD optimized at the M06 level are given in Table S8−S12, respectively.

Table S1. Computed S_r, D, and t indices in the electron excitation analysis of g-C_3N_4 and g-C_3N_4/CD at the optimized S_1 geometries.

**Compound**	$\boldsymbol{S_r}$	**D (Å)**	$\boldsymbol{S_r}$**/D (Å^{-1})**	$\boldsymbol{t}$ **(Å)**
g-C_3N_4	0.463	0.166	2.789	−1.071
g-C_3N_4/CD	0.691	3.242	0.213	0.379

Table S2. Efficiency comparison of g-C_3N_4/CD-10 and other reported photocatalysts for LEV degradation.[13]

Photocatalyst type	Photocatalyst	Dosage (g L^{-1})	Initial conc. (mg L^{-1})	Light source	Efficiency (%)	Time (min)	EEE* (mg (mg·kW·h)$^{-1}$)	Ref.
g-C_3N_4-based photocatalysts	Bi_2O_3/P-C_3N_4	1	10	500 W Xe lamp	89.2	75	1.4	a)
	VO-UCN@CCO	0.7	20	100 W LED lamp λ > 450 nm	95	120	13.6	b)
	HCN-$C_{0.5}$	1	10	300 W Xe lamp λ≥420 nm	100	20	10.1	c)
	ZIS/CPCN	0.4	25	300 W Xe lamp λ≥420 nm	86	180	6.0	d)
	Co_3O_4/Bi_2MoO_6@g-C_3N_4	1	25	300 W Xe lamp λ≥420 nm	95.21	60	7.9	e)
	0.5-CQD/BOI/CN	0.5	20	300 W Xe lamp	94.8	60	12.6	f)
	VO-WCN@CCS(30)	0.7	25	100 W LED lamp λ > 450 nm	98	120	17.5	g)
	**g-C_3N_4/CD-10**	0.5	10	10 W LED lamp λ=400-800 nm	95	120	**95.0**	**This work**
Other types of photocatalysts	$ZnFe_2O_4$/NCDs/Ag_2CO_3	0.6	10	300 W Xe lamp λ≥420 nm	88.75	90	3.3	h)
	Fe–ZnO/WO_3	0.5	10	30 W LED lamp λ=425-470 nm	96	180	21.3	i)
	Ag_3BiO_3/ZnO/BC	0.4	10	300 W Xe lamp	95.8	120	4.0	j)
	Cr-doped Cu_2O	1	40	500 W tungsten halogen lamp	79.6	240	1.6	k)

$Bi_4O_5I_2/Bi_3TaO_7$	0.5	5	300 W Xe lamp λ > 400 nm	92.23	60	3.1	l)
BTT-DATP-COF	0.1	10	300 W Xe lamp λ≥420 nm	100	100	20.0	m)
$TaON/Bi_2MoO_6$	0.4	20	300 W Xe lamp λ > 400 nm	92.7	75	12.4	n)

*EEE was calculated by the following equation:

Equivalent Environmental Efficiency (EEE) = (Initial conc.×Efficiency)/(Dosage×Light source power×Time)

Table S3. Hirshfeld charges and Fukui index of LEV computed at the M06/6-31G(d,p) level.

Label	Atom	q(N)	q(N+1)	q(N-1)	f^-	f^+	f^0
1	C	-0.0300	-0.0588	0.0168	0.0468	0.0288	0.0378
2	C	0.0387	0.0085	0.0615	0.0228	0.0302	0.0265
3	C	0.1269	0.0129	0.1411	0.0142	0.1140	0.0641
4	C	-0.0589	-0.1284	-0.0160	0.0430	0.0694	0.0562
5	C	0.0651	0.0163	0.1380	0.0729	0.0487	0.0608
6	N	0.0096	-0.0342	0.0208	0.0113	0.0438	0.0275
7	C	-0.0697	-0.1017	-0.0457	0.0240	0.0320	0.0280
8	O	-0.3213	-0.4284	-0.2991	0.0222	0.1070	0.0646
9	C	0.0968	0.0598	0.1298	0.0330	0.0370	0.0350
10	O	-0.1383	-0.1610	-0.0903	0.0480	0.0227	0.0354
11	C	0.0343	-0.0215	0.0787	0.0445	0.0558	0.0501
12	C	0.0546	0.0452	0.0596	0.0050	0.0095	0.0072
13	C	0.0813	-0.0091	0.0971	0.0157	0.0905	0.0531
14	C	0.2022	0.1870	0.2108	0.0086	0.0152	0.0119
15	F	-0.1010	-0.1215	-0.0786	0.0224	0.0204	0.0214
16	C	0.0481	0.0387	0.0601	0.0120	0.0094	0.0107
17	N	-0.0878	-0.1044	0.0655	0.1534	0.0165	0.0850
18	C	-0.0842	-0.0911	-0.0811	0.0031	0.0069	0.0050
19	O	-0.2319	-0.2499	-0.2233	0.0086	0.0180	0.0133
20	O	-0.3808	-0.4057	-0.3669	0.0139	0.0249	0.0194
21	C	-0.0066	-0.0141	0.0227	0.0293	0.0075	0.0184
22	C	-0.0089	-0.0138	0.0174	0.0263	0.0049	0.0156
23	C	-0.0114	-0.0140	-0.0013	0.0101	0.0026	0.0064
24	C	-0.0111	-0.0141	0.0016	0.0127	0.0030	0.0079
25	N	-0.1300	-0.1309	-0.1257	0.0043	0.0009	0.0026
26	C	-0.0438	-0.0450	-0.0386	0.0052	0.0012	0.0032
27	H	0.0591	0.0279	0.0803	0.0212	0.0312	0.0262
28	H	0.0172	0.0144	0.0304	0.0133	0.0028	0.0080
29	H	0.0422	0.0382	0.0579	0.0156	0.0040	0.0098
30	H	0.0387	0.0331	0.0420	0.0033	0.0055	0.0044
31	H	0.0523	0.0439	0.0565	0.0041	0.0084	0.0063
32	H	0.0463	0.0398	0.0500	0.0037	0.0065	0.0051
33	H	0.0723	0.0584	0.0807	0.0084	0.0139	0.0111
34	H	0.0806	0.0443	0.0900	0.0093	0.0363	0.0228
35	H	0.0547	0.0444	0.0682	0.0135	0.0103	0.0119
36	H	0.0700	0.0590	0.0810	0.0110	0.0110	0.0110
37	H	0.1369	0.1254	0.1406	0.0038	0.0115	0.0076
38	H	0.0279	0.0183	0.0766	0.0487	0.0096	0.0292
39	H	0.0367	0.0313	0.0587	0.0220	0.0055	0.0137
40	H	0.0446	0.0387	0.0673	0.0227	0.0059	0.0143
41	H	0.0229	0.0157	0.0681	0.0452	0.0073	0.0262
42	H	0.0426	0.0390	0.0567	0.0141	0.0036	0.0089

43	H	0.0162	0.0135	0.0290	0.0128	0.0027	0.0078
44	H	0.0381	0.0370	0.0426	0.0045	0.0011	0.0028
45	H	0.0381	0.0370	0.0426	0.0045	0.0011	0.0028
46	H	0.0214	0.0203	0.0263	0.0049	0.0011	0.0030

Table S4. The information of degradation compounds of LEV.

Compounds	Formula	m/z	Proposed structure
LEV	$C_{18}H_{20}O_4FN_4$	362.1544	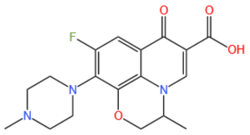
TP348	$C_{17}H_{18}O_4FN_3$	348.13711	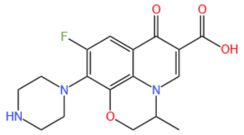
TP287	$C_{15}H_{17}O_3N_3$	287.1630	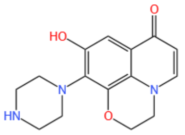
TP394	$C_{18}H_{20}O_6FN_3$	394.3311	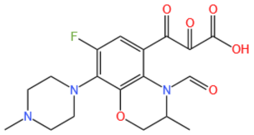
TP365	$C_{17}H_{20}O_5FN_3$	365.1353	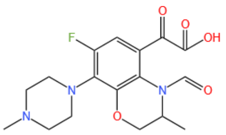
TP337	$C_{16}H_{20}O_4FN_3$	337.0764	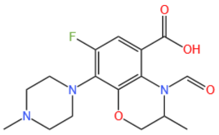
TP336	$C_{16}H_{18}O_4FN_3$	336.1369	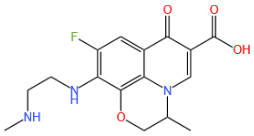
TP297	$C_{13}H_{11}O_4FN_2$	297.0796	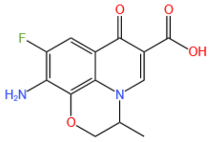
TP261	$C_{13}H_{12}O_4N_2$	261.0684	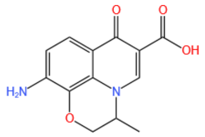
TP167	$C_7H_5O_4N_2$	167.0741	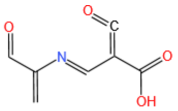

TP161	$C_9H_8ON_2$	161.0974	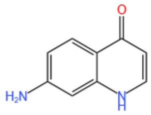
TP391	$C_{18}H_{19}O_6FN_3$	391.2851	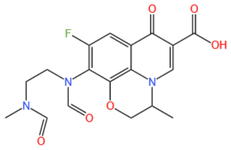
TP363	$C_{17}H_{18}O_5FN_3$	363.1559	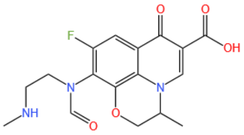
TP319	$C_{16}H_{18}O_3FN_3$	319.1651	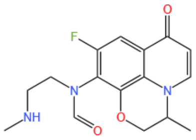
TP277	$C_{14}H_{16}O_2FN_3$	277.1812	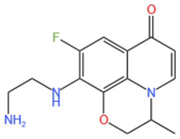
TP318	$C_{17}H_{20}O_2FN_3$	318.1627	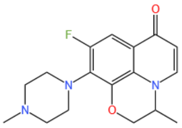
TP289	$C_{15}H_{16}O_2FN_3$	289.0990	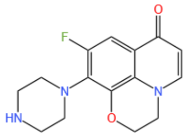
TP359	$C_{18}H_{21}O_5N_3$	359.2271	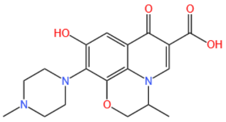
TP392	$C_{18}H_{21}O_7N_3$	392.1277	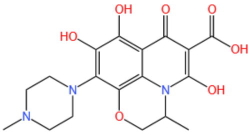
TP393	$C_{18}H_{23}O_7N_3$	393.1306	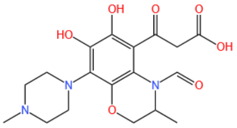
TP301	$C_{16}H_{19}O_3N_3$	301.1426	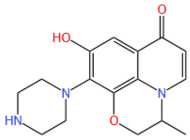

Table S5. Characteristics of various water samples.

Index	Tap water [a] (TW)	Sewage water [b] (SW)	Lake water [c] (LW)	River water [d] (RW)
pH	7.48	7.70	7.54	7.91
DOC (mg L^{-1}) [e]	5.5	14.1	36.1	21.1
Cu^{2+} (μg L^{-1})	9.3	4.1	3.3	11.8
Mg^{2+} (mg L^{-1})	1.2	1.3	0.6	1.3
Na^{+} (mg L^{-1})	2.1	4.0	4.1	7.2
K^{+} (mg L^{-1})	3.8	1.5	5.0	9.2
CO_3^{2-} (mg L^{-1})	n.d.	n.d.	n.d.	n.d.
SO_4^{2-} (mg L^{-1})	n.d.	n.d.	n.d.	n.d.
HCO_3^{-} (mg L^{-1})	46	75	81	95
NO_3^{-} (mg L^{-1})	n.d.	n.d.	n.d.	n.d.
Cl^{-} (mg L^{-1})	1505.8	1537.4	1244.7	2403.3

[a] Tap water sourced from our own lab located at F Building, Tsinghua Shenzhen International School

[b] Sewage water sourced from the effluent of secondary sedimentation tank of a local sewage water treatment plant in Nanshan District, Shenzhen City, China

[c] Lake water sourced from the campus of Tsinghua Shenzhen International School

[d] River water sourced from Dasha River in Nanshan District, Shenzhen City, China

[e] Dissolved organic carbon

*n.d.: not detected.

Table S6. LC settings for the determination of LEV intermediates.

Mobile phase	Column temp (°C)	Gradient (v/v)	Flow rate (mL/min)	Injection volume (μL)
A: 0.1% formic acid<br>B: methanol	40	0–1 min: A: B=90: 10<br>1–10 min: A: B=10: 90<br>10–11 min: A: B=0: 100<br>11–13 min: A: B=90: 10	0.3	5

Table S7. HPLC settings of different antibiotics.

Antibiotic	Detection wavelength (nm)	Column temp (°C)	Mobile phase (v/v)	Flow rate (mL/min)	Injection volume (μL)
Levofloxacin (LEV)	294	40	0.1% formic solution: acetonitrile = 74:26	1	10
Ofloxacin (OFL)	294	30	0.1% formic solution: methanol = 75:25	1	10
Ciprofloxacin (CIP)	273	30	0.1% formic solution: acetonitrile = 80:20	1	10
Norfloxacin (NOR)	274	30	0.1% formic solution: acetonitrile = 70:30	1	10
Tetracycline (TC)	268	30	0.1% formic solution: acetonitrile = 75:25	1	10
Oxytetracycline (OTC)	360	35	0.2% formic solution: methanol = 75:25	1	20

Table S8. Cartesian coordinates of the geometry of LEV optimized at the M06/6-31G(d,p) level.

Energy = −1262.26017020 Hartrees

#P M06/6-31g(d,p) opt freq nosymm scrf=(smd,solvent=water) integral(grid=ultrafine) scf=(xqc,maxcycle=1000)

1	C	3.630141	3.837152	0.736194
2	C	4.187928	3.039087	1.751061
3	C	4.267693	3.924376	-0.570079
4	C	2.470893	4.566502	1.011469
5	C	3.563142	2.941624	3.005166
6	N	5.357200	2.324665	1.502657
7	C	5.474906	3.157969	-0.729947
8	O	3.780852	4.633178	-1.486705
9	C	1.914952	4.504663	2.263612
10	O	4.054224	2.126011	3.976673
11	C	2.416518	3.695634	3.304537
12	C	5.986495	1.592113	2.623685
13	C	5.961373	2.405171	0.316605
14	C	6.239925	3.146905	-1.988425
15	F	0.839277	5.267433	2.508037
16	C	4.866978	1.058292	3.484788
17	N	1.836195	3.572045	4.563878
18	C	6.948026	2.491234	3.373699
19	O	5.752247	3.893530	-2.979982
20	O	7.266934	2.503401	-2.149288
21	C	2.623313	4.035851	5.710867
22	C	0.414022	3.840427	4.760040
23	C	2.127328	3.371109	6.973039
24	C	-0.042082	3.162531	6.034125
25	N	0.709641	3.641867	7.190044
26	C	0.261254	2.943441	8.380861
27	H	2.031838	5.208975	0.254579
28	H	0.067107	2.063582	5.924181
29	H	-1.107958	3.370324	6.191249
30	H	6.429073	3.353743	3.806693
31	H	7.420455	1.928234	4.185056
32	H	7.732013	2.858497	2.703610
33	H	6.517682	0.741171	2.183048
34	H	6.880744	1.833317	0.208327
35	H	4.238815	0.358825	2.917619
36	H	5.270293	0.549564	4.362995
37	H	4.918313	4.335905	-2.654840
38	H	2.534847	5.132608	5.805226
39	H	3.678945	3.797866	5.557812
40	H	-0.150930	3.443703	3.911081
41	H	0.210455	4.921080	4.832375
42	H	2.699012	3.741887	7.832894
43	H	2.313313	2.278722	6.897261
44	H	0.821032	3.290280	9.256571
45	H	-0.802846	3.137283	8.555704
46	H	0.398825	1.847627	8.301749

Table S9. Cartesian coordinates of the geometry of g-C_3N_4 optimized at the M06/6-31G(d,p) level.

Energy = −2238.26450840 Hartrees

#P M06/6-31g(d,p) opt freq nosymm scrf=(smd,solvent=water) integral(grid=ultrafine) scf=(xqc,maxcycle=1000)

1	C	-2.303664	-1.700402	-0.088282	43	C	3.536043	-4.868415	-1.915517
2	C	-2.241235	0.707595	0.135919	44	C	2.971538	-2.719428	-2.849932
3	C	-4.163699	-0.481523	0.394295	45	C	5.196532	-3.604805	-3.132877
4	C	-0.201709	-0.524829	-0.181533	46	C	4.622288	-1.524514	-3.823804
5	C	-0.240135	1.732960	-0.172012	47	N	1.742573	-2.855192	-2.374938
6	N	-1.589213	-0.505041	-0.064533	48	N	3.325678	-1.646465	-3.534664
7	N	-3.548825	0.704924	0.384373	49	N	2.338013	-4.925420	-1.349611
8	N	-1.558893	1.838818	0.042112	50	N	3.908331	-3.726540	-2.617361
9	N	-3.607576	-1.677585	0.149354	51	N	5.562092	-2.463997	-3.707399
10	N	0.472383	0.614383	-0.148505	52	C	-0.363001	-5.198554	-0.630921
11	C	-5.619276	-0.486421	0.672620	53	H	-0.706078	-5.228090	0.404597
12	H	-6.158475	-0.704992	-0.257535	54	H	-1.216646	-5.336462	-1.299112
13	H	-5.868065	-1.284173	1.378586	55	H	0.355982	-5.998616	-0.792183
14	H	-5.955270	0.478196	1.055492	56	C	6.234390	-0.302635	-5.211785
15	N	-1.669129	-2.831828	-0.376490	57	H	7.145132	-0.324069	-4.606791
16	N	0.421954	-1.676474	-0.342057	58	H	6.216645	-1.170977	-5.870331
17	C	-0.346780	-2.749046	-0.543564	59	H	6.219797	0.603083	-5.814018
18	N	0.409475	2.938002	-0.453761	60	N	4.401713	-5.871144	-1.800229
19	C	-0.320110	4.179612	-0.164059	61	N	6.042125	-4.618135	-3.010545
20	H	0.401451	4.987265	-0.054978	62	C	5.601999	-5.708000	-2.365765
21	H	-1.029478	4.433478	-0.956407	63	C	6.563755	-6.829199	-2.250936
22	H	-0.857615	4.052897	0.773854	64	H	7.030561	-7.026957	-3.220647
23	C	1.470236	3.013287	-1.355828	65	H	7.368905	-6.539749	-1.564536
24	C	3.085678	1.952363	-2.526663	66	H	6.082064	-7.731321	-1.871682
25	C	2.439862	4.212275	-3.022292					
26	C	4.014226	3.058063	-4.454077					
27	C	3.486988	5.251019	-4.754773					
28	N	1.560983	4.167188	-2.032526					
29	N	2.275611	1.965400	-1.482214					
30	N	3.164190	3.070872	-3.350685					
31	N	2.625900	5.321050	-3.735353					
32	N	4.170882	4.169692	-5.158884					
33	C	3.704281	6.486144	-5.544715					
34	H	4.776277	6.693057	-5.626826					
35	H	3.333574	6.332496	-6.565066					
36	H	3.191804	7.340154	-5.100241					
37	N	3.811293	0.884791	-2.806323					
38	N	4.625980	1.928379	-4.790440					
39	C	4.453423	0.885098	-3.974283					
40	N	0.297862	-3.918349	-0.905412					
41	N	5.041236	-0.311986	-4.358285					
42	C	1.523546	-3.889153	-1.565759					

Table S10. Cartesian coordinates of the geometry of g-C_3N_4/CD optimized at the M06/6-31G(d,p) level.

Energy = −5838.89489745 Hartrees

#P M06/6-31g(d,p) opt freq nosymm scrf=(smd,solvent=water) integral(grid=ultrafine) scf=(xqc,maxcycle=1000)

1	C	-1.511231	-0.460367	0.124389	46	H	5.655332	-0.321515	-6.837240
2	C	-3.726610	-0.727362	-0.221703	47	N	0.478166	-5.742857	-7.737222
3	C	-0.048798	-2.091672	-0.777716	48	N	2.802577	-5.213519	-7.840427
4	C	0.738602	-0.269955	0.251639	49	C	1.747607	-5.993281	-8.119248
5	N	-1.294542	-1.526324	-0.732244	50	C	-1.233400	-3.373034	-5.513864
6	N	-2.734323	-0.141011	0.495709	51	H	-1.764475	-2.655731	-6.164301
7	N	-0.446709	0.247965	0.530492	52	C	-0.819425	-3.575539	-2.298673
8	N	-3.631385	-1.539248	-1.227658	53	H	-0.634551	-4.417846	-2.962543
9	N	0.976742	-1.512602	-0.202222	54	C	-2.459402	-2.329098	-1.161381
10	N	0.127502	-3.244642	-1.467807	55	H	-2.594116	-3.133085	-0.411396
11	N	1.854167	0.541284	0.473813	56	N	-2.047586	-2.996718	-2.417509
12	C	1.754148	1.613184	1.468195	57	C	-2.907065	-3.654755	-3.372882
13	H	2.737009	1.780237	1.906449	58	C	-4.109788	-4.372602	-2.954175
14	H	1.396895	2.544146	1.017443	59	C	-2.344783	-3.943224	-4.610214
15	H	1.064307	1.300096	2.250094	60	C	-4.513646	-5.480444	-3.728296
16	C	2.866903	0.591819	-0.471744	61	C	-4.916276	-4.137558	-1.807455
17	C	3.660057	-0.133153	-2.466600	62	C	-2.786752	-5.103652	-5.298086
18	C	4.796028	1.551803	-1.181885	63	C	-5.433528	-6.362356	-3.166741
19	C	5.659759	0.860057	-3.343879	64	C	-3.788323	-5.934730	-4.856400
20	C	6.713157	2.413955	-2.055367	65	C	-5.902942	-4.993589	-1.307773
21	N	3.870292	1.448819	-0.235022	66	H	-4.808122	-3.180729	-1.325542
22	N	2.720194	-0.176164	-1.543781	67	C	-6.016050	-6.293379	-1.907226
23	N	4.708401	0.770090	-2.330172	68	C	-1.863319	-5.578631	-6.242603
24	N	5.822409	2.388648	-1.055997	69	C	-1.842128	-6.896400	-6.648565
25	N	6.681416	1.696188	-3.185560	70	H	-1.027513	-7.240808	-7.260240
26	C	7.854873	3.350036	-1.913848	71	C	-2.796064	-7.813621	-6.126005
27	H	8.792263	2.842165	-2.160510	72	C	-3.828934	-7.280443	-5.316955
28	H	7.738557	4.168991	-2.634124	73	C	-4.709186	-8.145801	-4.647316
29	H	7.906235	3.768082	-0.907600	74	C	-2.629147	-9.262861	-6.228324
30	N	3.597832	-0.923276	-3.515223	75	C	-4.503360	-9.501429	-4.712122
31	N	5.524653	0.129712	-4.441568	76	C	-5.046216	-10.360318	-3.726935
32	C	4.480159	-0.717309	-4.490591	77	C	-5.864876	-9.885976	-2.694514
33	N	4.349464	-1.454114	-5.645358	78	H	-6.253440	-10.538279	-1.919541
34	C	0.283841	-4.736488	-6.944904	79	C	-6.056857	-8.472675	-2.627191
35	C	1.052584	-2.848777	-5.619938	80	C	-5.519708	-7.674489	-3.626389
36	C	2.574938	-4.140055	-7.080805	81	N	-6.439353	-7.601216	-1.587292
37	C	3.261478	-2.319657	-5.907173	82	C	-6.899652	-8.078283	-0.293937
38	N	-0.076940	-2.710350	-5.013019	83	H	-6.678790	-7.337029	0.477371
39	N	2.098475	-2.032263	-5.373268	84	H	-7.974402	-8.289964	-0.291950
40	N	-0.913381	-4.555404	-6.383493	85	H	-6.361380	-8.997681	-0.039158
41	N	1.281722	-3.852483	-6.616008	86	N	1.869694	-7.136192	-8.813475
42	N	3.571511	-3.347759	-6.722480	87	C	3.125405	-7.678475	-9.299177
43	C	5.430597	-1.368686	-6.634855	88	H	3.855198	-6.874951	-9.405738

44	H	6.336074	-1.863159	-6.271673
45	H	5.104375	-1.849400	-7.552901
91	C	0.663384	-7.942267	-8.947697
92	C	-0.768585	-9.482648	-8.042907
93	C	-1.224084	-8.284921	-10.147231
94	C	-2.486019	-10.185083	-9.404466
95	C	-3.078567	-9.030154	-11.253697
96	N	0.497605	-8.853032	-8.035190
97	N	-0.082680	-7.605395	-10.026611
98	N	-2.022121	-8.181323	-11.210984
99	N	-1.613794	-9.129590	-9.145519
100	N	-3.345750	-10.049347	-10.444778
101	C	-4.015114	-8.803121	-12.389018
102	H	-4.408500	-7.781111	-12.348771
103	H	-3.479791	-8.896858	-13.340633
104	H	-4.844655	-9.512425	-12.370212
105	N	-1.430210	-9.754357	-6.783948
106	N	-2.334016	-11.205647	-8.609517
107	C	-1.265957	-10.865724	-7.716235
108	H	-0.650891	-11.685366	-7.357182
109	N	-5.010950	-0.324834	0.227623
110	C	-5.103568	0.987583	0.785716
111	C	-5.104710	3.207534	0.385415
112	C	-5.338490	2.286478	2.609557
113	C	-5.389386	4.672336	2.277979
114	C	-5.152363	5.468089	0.157835
115	N	-5.036795	4.255401	-0.412213
116	N	-5.024680	1.964348	-0.099766
117	N	-5.327318	5.717399	1.454385
118	N	-5.280613	3.385298	1.756052
119	N	-5.235192	1.062398	2.101639
120	N	-5.549152	4.838166	3.582017
121	N	-5.486344	2.473911	3.914356
122	C	-5.585643	3.736333	4.343118
123	C	-5.076927	6.625217	-0.762069
124	H	-4.127492	6.597796	-1.308170
125	H	-5.872269	6.547766	-1.511981
126	H	-5.167654	7.569985	-0.225199
127	C	-5.756245	3.952564	5.797718
128	H	-4.955826	4.601968	6.168907
129	H	-6.698995	4.481011	5.979362
130	H	-5.752602	3.010001	6.345962
131	C	-6.063656	-1.205194	0.436367
132	C	-8.177633	-1.519413	1.150145
133	C	-6.880721	-3.289628	0.182843
134	C	-9.136136	-3.762741	1.032429
135	N	-5.826511	-2.508195	0.226725
89	H	3.520314	-8.435332	-8.612031
90	H	2.956942	-8.139129	-10.276984
136	N	-6.881582	-4.529964	-0.351957
137	N	-7.202058	-0.659899	0.822158
138	N	-8.087570	-2.841817	0.713236
139	N	-9.158826	-4.919819	0.485506
140	N	-9.207406	-1.118755	1.857786
141	N	-10.061741	-3.352691	1.935725
142	C	-10.072100	-2.083512	2.272972
143	C	-11.126853	-1.615958	3.207338
144	H	-10.666060	-1.158389	4.089818
145	H	-11.724480	-0.834615	2.723951
146	H	-11.778682	-2.434800	3.515531
147	C	-8.255923	-5.063925	-0.609548
148	H	-8.636023	-4.541792	-1.502234
149	H	-8.162229	-6.113508	-0.865424
150	C	-3.495448	-10.081539	-5.509318
151	C	-2.846802	-13.634215	-4.904006
152	C	-4.357526	-15.137952	-3.728120
153	C	-2.434266	-15.883351	-4.862090
154	H	-1.834765	-16.759372	-5.094353
155	H	-3.828504	-17.097651	-3.908697
156	N	-2.594473	-12.472354	-5.362921
157	N	-2.014208	-14.706662	-5.153794
158	N	-4.354673	-11.590389	-3.903749
159	N	-4.023479	-13.903748	-4.145642
160	N	-3.624002	-16.155012	-4.232964
161	N	-5.278512	-15.381018	-2.805587
162	C	-5.403825	-14.401989	-1.893030
163	C	-5.871891	-14.768033	-0.541753
164	H	-5.000835	-14.949297	0.101759
165	H	-6.468262	-13.972704	-0.087464
166	H	-6.443739	-15.695742	-0.597385
167	C	-4.907364	-12.857021	-3.591317
168	H	-5.908584	-13.023829	-4.025585
169	N	-5.023023	-13.150172	-2.152135
170	C	-4.562218	-12.263893	-1.076019
171	H	-4.005647	-11.437938	-1.516299
172	H	-5.405268	-11.873708	-0.498398
173	H	-3.893506	-12.816231	-0.411755
174	C	-3.691455	-11.560273	-5.265383
175	H	-4.450559	-11.894626	-6.001951

Table S11. Cartesian coordinates of the geometry of LEV-g-C_3N_4/CD optimized at the M06/6-31G(d,p) level.

#P M06/6-31G(d,p) opt=tight freq scrf=(smd,solvent=water) nosymm integral(grid=ultrafine) scf=(xqc,maxcycle=1000)

Energy = −7101.19866089 Hartrees

1	C	-1.569440	0.126514	-0.333956	46	H	6.734566	-1.520199	-5.873768
2	C	-3.758619	-0.408603	-0.536181	47	N	0.473543	-5.448879	-8.030557
3	C	0.018286	-1.456693	-1.114950	48	N	2.827275	-5.077682	-8.138392
4	C	0.646544	0.575860	-0.396401	49	C	1.716916	-5.756558	-8.457052
5	N	-1.276429	-1.018798	-1.056573	50	C	-0.993500	-3.272645	-5.470489
6	N	-2.808770	0.356676	0.061035	51	H	-1.409090	-2.415202	-6.028237
7	N	-0.575213	0.983226	-0.068437	52	C	-0.655355	-3.258156	-2.291987
8	N	-3.608267	-1.331120	-1.434872	53	H	-0.432879	-4.200012	-2.787400
9	N	1.004077	-0.675172	-0.757427	54	C	-2.361858	-1.984859	-1.306406
10	N	0.275875	-2.709106	-1.565452	55	H	-2.388351	-2.679145	-0.443770
11	N	1.676991	1.477301	-0.278922	56	N	-1.918954	-2.780062	-2.469621
12	C	1.527664	2.659025	0.569781	57	C	-2.725132	-3.531230	-3.389463
13	H	2.489103	3.167398	0.622972	58	C	-3.908655	-4.268311	-2.956732
14	H	0.786057	3.339407	0.143610	59	C	-2.148312	-3.832941	-4.618373
15	H	1.217062	2.370177	1.576431	60	C	-4.296351	-5.387715	-3.718960
16	C	2.869962	1.225795	-0.962745	61	C	-4.718829	-4.039072	-1.811217
17	C	3.814318	0.232001	-2.754546	62	C	-2.602542	-4.977189	-5.318239
18	C	5.121290	1.066339	-0.900632	63	C	-5.186389	-6.287949	-3.138539
19	C	6.170202	-0.222180	-2.654823	64	C	-3.583373	-5.823595	-4.859953
20	C	7.372628	0.754090	-0.987467	65	C	-5.702273	-4.900517	-1.311018
21	N	4.001712	1.477442	-0.310238	66	H	-4.627360	-3.075369	-1.340290
22	N	2.733934	0.771020	-2.206080	67	C	-5.773269	-6.223937	-1.881203
23	N	5.033173	0.340913	-2.086915	68	C	-1.721096	-5.417047	-6.316108
24	N	6.310508	1.287805	-0.362663	69	C	-1.703879	-6.723688	-6.756470
25	N	7.355313	-0.004840	-2.085180	70	H	-0.916182	-7.044490	-7.415293
26	C	8.691031	1.022107	-0.366176	71	C	-2.617463	-7.667470	-6.204080
27	H	9.509231	0.683833	-1.003222	72	C	-3.609184	-7.164106	-5.329402
28	H	8.795283	2.090420	-0.152178	73	C	-4.433989	-8.050529	-4.625572
29	H	8.746730	0.499550	0.596438	74	C	-2.464254	-9.111323	-6.362672
30	N	3.745184	-0.415000	-3.903521	75	C	-4.214090	-9.398366	-4.717616
31	N	6.053524	-0.980000	-3.731367	76	C	-4.724534	-10.281973	-3.737757
32	C	4.834377	-1.083415	-4.284714	77	C	-5.519269	-9.831634	-2.673623
33	N	4.747475	-1.940313	-5.369160	78	H	-5.894002	-10.505946	-1.909748
34	C	0.377157	-4.550465	-7.101462	79	C	-5.731206	-8.417950	-2.581838
35	C	1.346200	-3.031534	-5.468560	80	C	-5.222827	-7.605571	-3.580881
36	C	2.697452	-4.098746	-7.238893	81	N	-6.136948	-7.551033	-1.545070
37	C	3.578879	-2.625827	-5.749192	82	C	-6.556427	-8.046019	-0.244086
38	N	0.224740	-2.851926	-4.861934	83	H	-6.341121	-7.301116	0.525685
39	N	2.500233	-2.506366	-5.007234	84	H	-7.623328	-8.293329	-0.220798
40	N	-0.788795	-4.376969	-6.470261	85	H	-5.984138	-8.949169	-0.006411
41	N	1.446174	-3.799164	-6.680239	86	N	1.757741	-6.850175	-9.239825
42	N	3.749268	-3.413133	-6.832006	87	C	3.001353	-7.458637	-9.684803
43	C	5.999248	-2.299814	-6.053074	88	H	3.706084	-6.682194	-9.986941

44	H	6.390008	-3.255236	-5.690392	89	H	3.455556	-8.068134	-8.895068
45	H	5.810402	-2.376162	-7.121256	90	H	2.785915	-8.093362	-10.547537
91	C	0.536466	-7.644782	-9.300975	140	N	-9.280558	-0.938760	1.429693
92	C	-0.796140	-9.246237	-8.345506	141	N	-10.066410	-3.188580	1.624766
93	C	-1.465257	-7.929531	-10.316129	142	C	-10.137749	-1.900479	1.865746
94	C	-2.645948	-9.881989	-9.562992	143	C	-11.259952	-1.404608	2.701747
95	C	-3.396610	-8.649821	-11.304963	144	H	-10.865984	-0.875014	3.576324
96	N	0.464050	-8.610322	-8.432978	145	H	-11.848315	-0.676312	2.131932
97	N	-0.319036	-7.250178	-10.273816	146	H	-11.906366	-2.221084	3.027199
98	N	-2.362552	-7.774552	-11.292487	147	C	-8.044459	-5.077538	-0.627870
99	N	-1.750576	-8.837476	-9.335181	148	H	-8.401781	-4.743612	-1.616005
100	N	-3.588179	-9.710496	-10.525022	149	H	-7.869400	-6.145625	-0.693155
101	C	-4.382090	-8.428726	-12.400721	150	C	-3.268533	-9.954800	-5.597979
102	H	-4.690532	-7.378189	-12.435054	151	C	-2.581284	-13.518510	-5.119438
103	H	-3.903522	-8.646624	-13.363442	152	C	-3.954117	-15.051583	-3.819540
104	H	-5.257437	-9.072492	-12.291135	153	C	-2.132449	-15.762316	-5.129425
105	N	-1.323099	-9.583367	-7.040402	154	H	-1.542960	-16.627374	-5.420945
106	N	-2.421018	-10.938976	-8.835672	155	H	-3.407002	-17.000234	-4.048872
107	C	-1.264509	-10.645176	-8.041035	156	N	-2.374962	-12.349197	-5.581681
108	H	-0.621432	-11.483405	-7.789797	157	N	-1.760656	-14.577810	-5.452202
109	N	-5.064860	-0.076007	-0.100151	158	N	-4.046972	-11.505229	-3.991388
110	C	-5.241873	1.248396	0.413048	159	N	-3.689903	-13.811108	-4.270155
111	C	-5.387122	3.451509	-0.044264	160	N	-3.253738	-16.055494	-4.393921
112	C	-5.489740	2.581703	2.210211	161	N	-4.771344	-15.312543	-2.809493
113	C	-5.648916	4.953994	1.822367	162	C	-4.828581	-14.333462	-1.889118
114	C	-5.538268	5.701676	-0.325440	163	C	-5.155056	-14.705063	-0.497959
115	N	-5.402312	4.479399	-0.870018	164	H	-4.222629	-14.834950	0.067200
116	N	-5.264670	2.200681	-0.500820	165	H	-5.745887	-13.931912	0.000265
117	N	-5.661945	5.978874	0.971399	166	H	-5.685381	-15.658494	-0.493751
118	N	-5.509194	3.659028	1.328292	167	C	-4.538552	-12.783202	-3.632758
119	N	-5.338995	1.351529	1.730312	168	H	-5.574185	-12.975811	-3.963272
120	N	-5.766428	5.147632	3.126988	169	N	-4.505406	-13.075623	-2.186166
121	N	-5.608581	2.796167	3.513854	170	C	-4.018541	-12.153049	-1.154929
122	C	-5.742010	4.064448	3.914930	171	H	-3.522813	-11.313904	-1.640028
123	C	-5.549563	6.835921	-1.276366	172	H	-4.841416	-11.786710	-0.534069
124	H	-4.620005	6.836068	-1.856462	173	H	-3.289316	-12.666387	-0.524283
125	H	-6.366811	6.703531	-1.994134	174	C	-3.458993	-11.439930	-5.389992
126	H	-5.662715	7.789808	-0.760276	175	H	-4.263482	-11.747067	-6.089897
127	C	-5.874174	4.311086	5.368686	176	C	-7.839717	-7.711151	-5.418627
128	H	-5.054504	4.956112	5.704872	177	C	-7.123420	-7.253284	-6.537539
129	H	-6.803801	4.856313	5.565586	178	C	-7.932483	-9.129704	-5.125838
130	H	-5.864517	3.379584	5.935461	179	C	-8.443016	-6.772182	-4.571985
131	C	-6.081136	-0.995285	0.132119	180	C	-7.024052	-5.875654	-6.819264
132	C	-8.204705	-1.350587	0.802001	181	N	-6.460489	-8.171565	-7.349253
133	C	-6.801730	-3.132795	0.014673	182	C	-7.206336	-10.002542	-6.010879
134	C	-9.072320	-3.632012	0.813338	183	O	-8.593135	-9.550465	-4.140171
135	N	-5.787830	-2.297876	0.017838	184	C	-8.314196	-5.443024	-4.854239
136	N	-6.719068	-4.417190	-0.402840	185	O	-6.369205	-5.452067	-7.934604
137	N	-7.250321	-0.475141	0.454505	186	C	-7.589425	-4.922658	-5.958052

138	N	-8.043911	-2.697783	0.470129
139	N	-9.021034	-4.833681	0.378913
189	C	-7.127029	-11.452993	-5.775893
190	F	-8.917459	-4.557183	-4.044286
191	C	-5.279749	-6.289982	-8.315978
192	N	-7.544199	-3.546165	-6.124213
193	C	-6.687337	-7.802867	-9.762283
194	O	-7.794607	-11.913416	-4.719101
195	O	-6.477366	-12.223581	-6.472316
196	C	-7.350996	-2.892251	-7.417184
197	C	-6.977571	-2.741986	-5.035042
198	C	-5.919251	-2.458378	-7.667929
199	C	-5.537354	-2.388648	-5.335796
200	N	-5.415312	-1.629937	-6.574680
201	C	-4.015240	-1.336901	-6.822902
202	H	-9.018579	-7.094908	-3.709117
203	H	-4.956098	-3.333969	-5.401000
204	H	-5.119263	-1.800193	-4.508799
205	H	-7.597140	-7.213357	-9.601926
206	H	-6.182437	-7.422440	-10.656293
207	H	-6.972796	-8.845649	-9.939763
208	H	-4.882685	-8.342982	-8.697794
209	H	-5.933024	-10.124226	-7.742608
210	H	-4.512514	-6.261859	-7.530865
211	H	-4.864091	-5.869199	-9.236286
212	H	-8.251936	-11.137921	-4.278225
213	H	-7.990898	-1.996734	-7.413533
214	H	-7.707803	-3.541133	-8.218636
215	H	-7.023569	-3.293201	-4.091046
216	H	-7.575450	-1.826646	-4.920661
217	H	-5.872239	-1.885552	-8.603301
218	H	-5.274135	-3.351857	-7.791912
219	H	-3.907913	-0.732939	-7.730884
220	H	-3.586939	-0.778418	-5.982199
221	H	-3.419081	-2.262118	-6.961895
187	C	-5.762507	-7.697637	-8.567793
188	C	-6.501147	-9.477821	-7.072844

Table S12. Cartesian coordinates of the geometry of LEV-g-C_3N_4 optimized at the M06/6-31G(d,p) level.

#P M06/6-31g(d,p) opt scrf=(smd,solvent=water) nosymm integral(grid=ultrafine) scf=(xqc,maxcycle=1000)

Energy = −3500.55399205 Hartrees

1	C	-1.204038	-0.135347	0.405057	46	H	6.519203	-2.255586	-6.077866
2	C	-3.437707	-0.560861	0.334501	47	N	1.216245	-6.091361	-6.274572
3	C	0.282410	-1.915026	-0.252295	48	N	3.371623	-5.314850	-6.972594
4	C	1.050250	0.089125	0.440859	49	C	2.227983	-5.986681	-7.146955
5	N	-1.007844	-1.402075	-0.138872	50	C	0.671068	-4.915135	-3.014948
6	N	-2.439019	0.279342	0.647573	51	C	-0.602674	-3.701510	-1.315877
7	N	-0.149912	0.639932	0.636525	52	C	-2.091764	-2.191537	-0.505781
8	N	-3.323879	-1.759454	-0.244449	53	N	-1.880273	-3.337963	-1.132671
9	N	1.306589	-1.188000	0.155692	54	C	4.232134	2.090663	-3.773842
10	N	0.464665	-3.112052	-0.788978	55	C	5.572831	2.481844	-3.950525
11	N	2.139512	0.934905	0.602562	56	C	3.648778	1.051768	-4.608253
12	C	1.942111	2.185637	1.346150	57	C	3.484179	2.679327	-2.749203
13	H	2.916252	2.619683	1.559319	58	C	6.169772	3.405883	-3.076568
14	H	1.348832	2.889888	0.757261	59	N	6.322677	1.920695	-4.982358
15	H	1.426680	1.977374	2.284536	60	C	4.520921	0.483789	-5.603501
16	C	3.344532	0.704291	-0.052208	61	O	2.464125	0.670073	-4.437442
17	C	4.455703	-0.373362	-1.702338	62	C	4.072989	3.613040	-1.937748
18	C	5.580478	1.086623	-0.153001	63	O	7.483333	3.740616	-3.185158
19	C	6.847305	-0.137110	-1.811616	64	C	5.422949	4.014538	-2.050040
20	C	7.813101	1.412891	-0.450541	65	C	7.672254	2.467994	-5.241883
21	N	4.423444	1.308892	0.457587	66	C	5.804569	0.963435	-5.754879
22	N	3.301866	-0.053359	-1.145538	67	C	4.099841	-0.634955	-6.463245
23	N	5.636943	0.167505	-1.197637	68	F	3.322310	4.178372	-0.979468
24	N	6.695088	1.703308	0.225741	69	C	8.285221	2.795198	-3.900353
25	N	7.947900	0.490475	-1.412619	70	N	6.023228	4.913143	-1.189730
26	C	9.020895	2.212796	-0.134520	71	C	7.592409	3.654324	-6.180871
27	H	9.931852	1.635258	-0.306926	72	O	2.895803	-1.149540	-6.210052
28	H	9.040578	3.077072	-0.817037	73	O	4.795132	-1.103607	-7.354362
29	H	8.991566	2.595419	0.888565	74	C	6.675718	6.115483	-1.699394
30	N	4.507093	-1.199263	-2.731971	75	C	5.623084	5.043222	0.205280
31	N	6.886004	-1.061190	-2.763644	76	C	7.872038	6.450767	-0.839568
32	C	5.712657	-1.569166	-3.156301	77	C	6.839205	5.405087	1.032716
33	N	5.763765	-2.558967	-4.133860	78	N	7.481540	6.626496	0.556188
34	C	1.365893	-5.492890	-5.099335	79	C	8.665573	6.883983	1.355611
35	C	2.777867	-4.305550	-3.555742	80	H	2.442218	2.409989	-2.601471
36	C	3.542075	-4.704999	-5.803714	81	H	7.552590	4.554378	1.009712
37	C	4.708410	-3.436147	-4.336440	82	H	6.536230	5.547485	2.077798
38	N	1.845298	-4.433048	-2.624165	83	H	7.002756	4.468339	-5.744783
39	N	3.916941	-3.691341	-3.295497	84	H	8.600581	4.029035	-6.384882
40	N	0.405419	-5.508857	-4.185404	85	H	7.131784	3.361124	-7.129745
41	N	2.548396	-4.806600	-4.835031	86	H	8.257317	1.658249	-5.692561
42	N	4.627581	-3.985501	-5.552494	87	H	6.458911	0.566750	-6.529639
43	C	6.873856	-2.580650	-5.095774	88	H	8.400140	1.885786	-3.291884

44	H	7.650337	-1.906079	-4.743125	89	H	9.262194	3.265718	-4.033564
45	H	7.280531	-3.590717	-5.172451	90	H	2.468409	-0.598474	-5.493745
91	H	5.961119	6.956936	-1.683410					
92	H	6.985725	5.963514	-2.735504					
93	H	5.224872	4.087847	0.562109					
94	H	4.844580	5.814098	0.330237					
95	H	8.339360	7.376333	-1.198341					
96	H	8.624665	5.638886	-0.939912					
97	H	9.167118	7.794066	1.008437					
98	H	8.391251	7.023100	2.407160					
99	H	9.394676	6.051451	1.301504					
100	C	2.038166	-6.671838	-8.447458					
101	H	2.953393	-6.663505	-9.040739					
102	H	1.702296	-7.700797	-8.285430					
103	H	1.243903	-6.163931	-9.007732					
104	N	-0.422698	-4.804354	-2.152148					
105	C	-4.806359	-0.084607	0.644702					
106	H	-4.854132	0.272540	1.678241					
107	H	-5.042936	0.772412	0.002709					
108	H	-5.547959	-0.867875	0.482951					
109	C	-1.613810	-5.600880	-2.476851					
110	H	-2.218917	-5.137385	-3.260639					
111	H	-1.289767	-6.585974	-2.807642					
112	H	-2.213681	-5.707774	-1.574937					

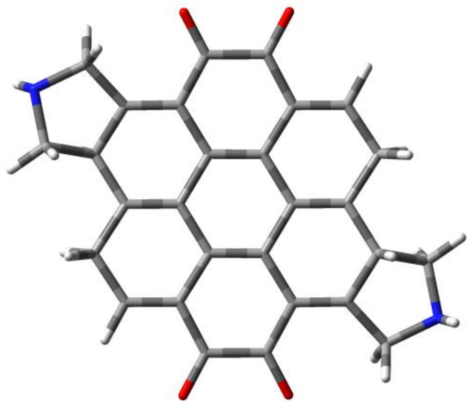

Figure S1. The optimized geometries of CD (The red element represents the oxygen atoms; the blue element represents the nitrogen atoms).

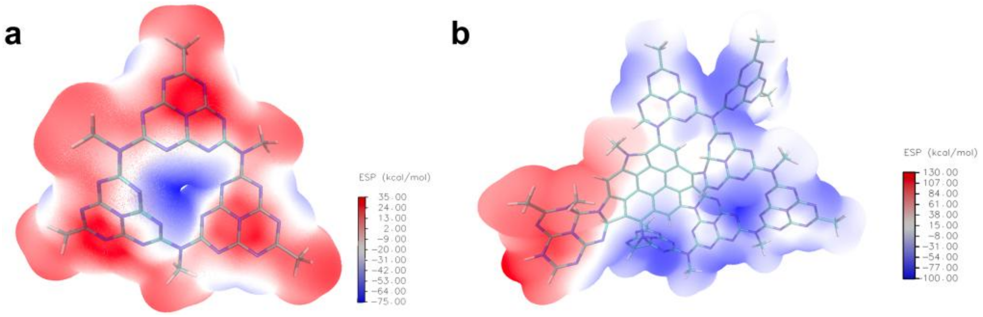

Figure S2. Electrostatic potential surface of (a) g-C_3N_4 and (b) g-C_3N_4/CD.

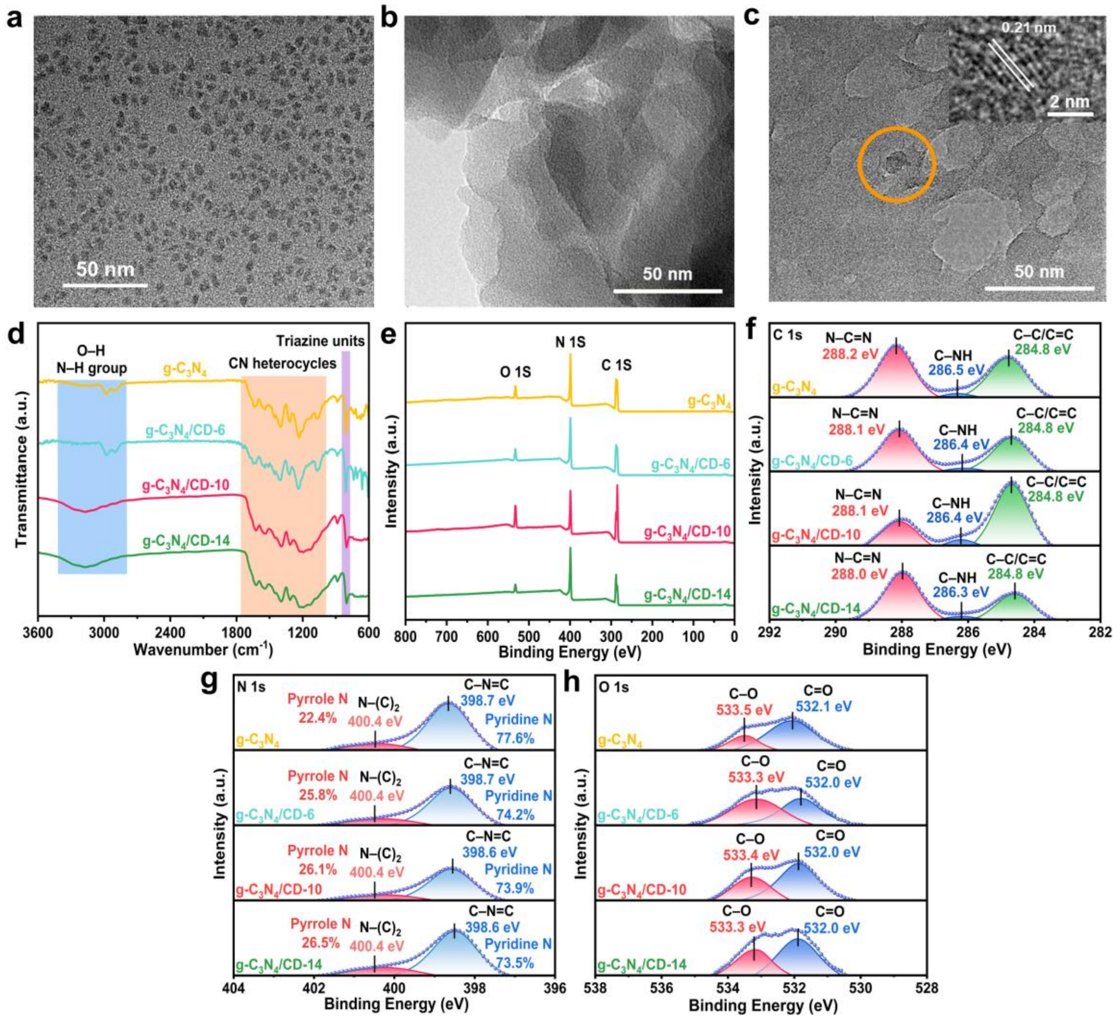

Figure S3. TEM image of (a) CD, (b) g-C_3N_4, and (c) g-C_3N_4/CD-10. (d) FT-IR spectra, (e) XPS survey spectra, and high-resolution XPS (f) C 1s, (g)N 1s, and (h) O 1s spectra of g-C_3N_4, g-C_3N_4/CD-6, g-C_3N_4/CD-10, and g-C_3N_4/CD-14.

Supplementary Note 1:

The microstructure of CD was characterized by TEM. The TEM images in Figure S3a showed that the CD are homogeneously dispersed without obvious agglomeration, presenting ellipsoidal particles with small particle sizes. Utilizing FT-IR spectroscopy (Figure S7a), the surface functional groups and structural attributes of CD were thoroughly examined. The results indicated that the CD primarily consist of an aromatic framework, with surface functionalities involving –OH, –NH, and –C=O groups. The C–N stretching vibration observed

at 1380 cm^{-1} served as a confirmatory signature of successful nitrogen doping.[14] Characterization by XPS determined the chemical state of elements of CD. As shown in Figure S7b three distinct peaks were observed at 284.4, 399.3, and 530.4 eV in the XPS spectrum, corresponding to the 1s orbitals of carbon, nitrogen, and oxygen atoms, respectively. The atomic content of the three elements in CD was estimated to be 66.72% C, 14.37% N, and 18.91% O. Specifically, the C 1s spectrum of CD (Figure S7c) could be deconvolved into three peaks at 284.5, 285.8 and 287.6 eV, which were ascribed to C–C/C=C bonds, C–N bonds and C–O/C=O bonds, respectively. For the fine spectra of N 1s (Fig. S7d), it was shown that CD synthesized from ammonium citrate and ethylenediamine contained pyrrole N with a binding energy of 399.7 eV (91.7% content) and graphite N with a binding energy of 401.3 eV (8.3%).[15] The results were in agreement with previous reports that ethylenediamine was employed as a dopant predominantly generate pyrrole N in CD.[16] In addition, the high-resolution O 1s spectrum of CD showed two peaks (Figure S7e), with C=O bonds at 530.9 eV and C–O bands at 532.9 eV. In short, the CD with N- and O-containing functional groups present on the surface were successfully synthesized.

The g-C_3N_4 and g-C_3N_4/CD were prepared by a facile thermal polymerization method. As observed from Figure S14a&b, both catalysts exhibited the appearance of scale-like aggregates, indicating that the doping of CD does not induce morphology change of g-C_3N_4. In addition, SEM/EDS elemental mapping showed that the distribution of O element in g-C_3N_4/CD was denser than that in g-C_3N_4 (the O content increased from 2.32% to 3.7%), possibly attributed to the doping of CD with oxygen-rich functional groups. On the other hand, the TEM images (Figure S3b&c) showed that the catalysts had a sheet-like morphology, while some black dots

were distributed on the g-C_3N_4/CD. The HRTEM image in Figure S3c inset demonstrated that the black dots exhibited clear lattice stripes (0.21 nm), proving the presence of CD. These results revealed that the synthesized CD could be embedded in g-C_3N_4 surface without disrupting its molecular structure.

The FT-IR spectra of different catalysts are shown in Figure S3d. It was clear that all samples showed almost the same IR spectra. The sharp peak near 810 cm^{-1} was ascribed to the out-of-plane bending vibrations of the tri-s-triazine units, while the broad absorption band around 1200–1600 cm^{-1} corresponds to the stretching vibration of the C–N heterocycles. Notably, the intensity of the absorption bands corresponding to O–H and N–H groups were significantly increased in g-C_3N_4/CD-10 and g-C_3N_4/CD-14, which may be attributed to the incorporation of a large number of CD containing hydroxyl- and amino-functional groups. These results demonstrated that the introduction of CD essentially does not alter the pristine crystalline structure of g-C_3N_4.

The surface structure and composition of the samples were further analyzed by XPS. Figure S3e shows that all samples exhibited three peaks belonging to C, N, and O elements. The C 1s high-resolution spectrum of g-C_3N_4 (Figure S3f) could be divided into three peaks centered at 284.8, 286.5, and 288.2 eV, corresponding to the C–C/C=C bonds, the C–NH bonds, and the basic tri-s-triazine unit (N–C=N) of g-C_3N_4, respectively.[17] The increased peak area of the C–NH bonds was also attributed to the introduction of CD with nitrogen-rich functional groups. In addition, the peak of the N–C=N bonds in the composites were all shifted towards lower binding energy compared to the g-C_3N_4, which was caused by the introduction of CD that changed the partial charge distribution.[18] For the N 1s high-resolution spectrum of g-C_3N_4

(Figure S3g), the XPS peaks at 398.7 and 400.4 eV are assigned to the N–C=N bonds and a sp^2-hybridized N atom (N–$(C)_2$), respectively.[19] It can be noticed that the relative content of pyrrole N in the composites gradually increases compared to the pure g-C_3N_4, further confirming the doping of the CD. The O 1s high-resolution spectrum of g-C_3N_4 (Figure S3h) exhibited two obvious peaks with binding energy of 532.1 and 533.5 eV, which were ascribed to the C–O and C=O bonds, respectively.[20] Compared to pure g-C_3N_4, the binding energy of C–O bonds was reduced, which might result from the presence of hydrogen bonds between hydroxyl groups in CD and amino groups in g-C_3N_4. In the composites, the binding energy of the C=O bond follows the same trend as that of the C−N bond, which might be due to the electrons of pyrrole N moving toward the C=O bonds, thus reducing the binding energy of C=O bonds.

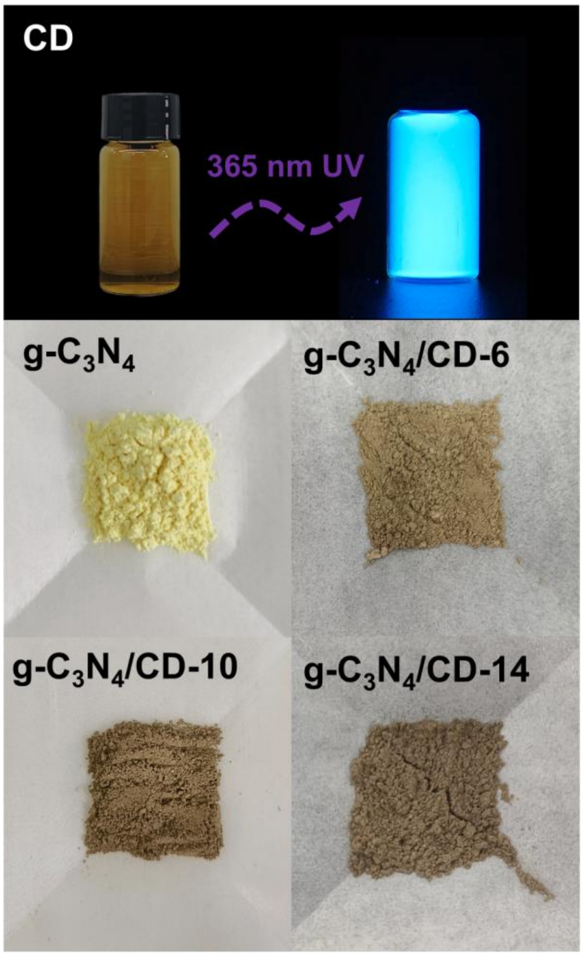

Figure S4. Photographs of the CD under sunlight and 365-nm UV irradiation, g-C_3N_4, g-C_3N_4/CD-6, g-C_3N_4/CD-10, and g-C_3N_4/CD-14.

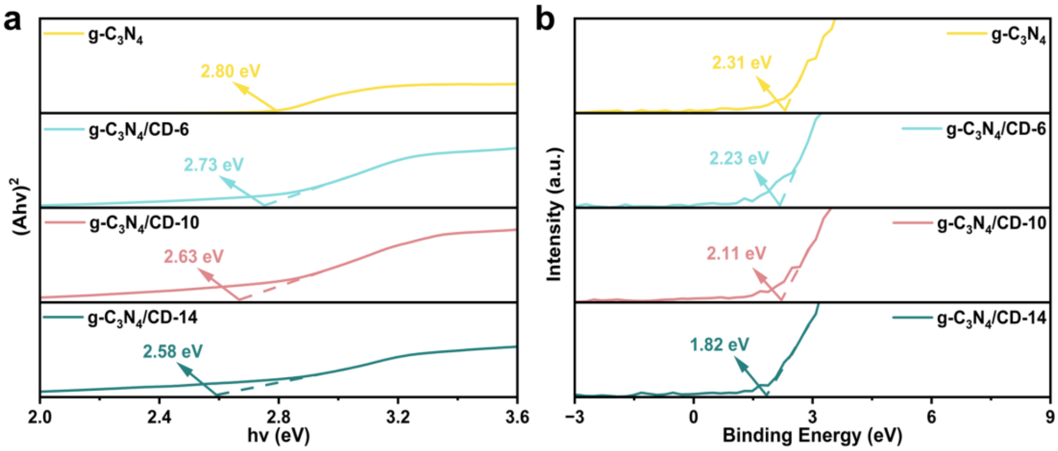

Figure S5. (a) Tauc plots, and (b) VB XPS plots of g-C_3N_4, g-C_3N_4/CD-6, g-C_3N_4/CD-10, and g-C_3N_4/CD-14.

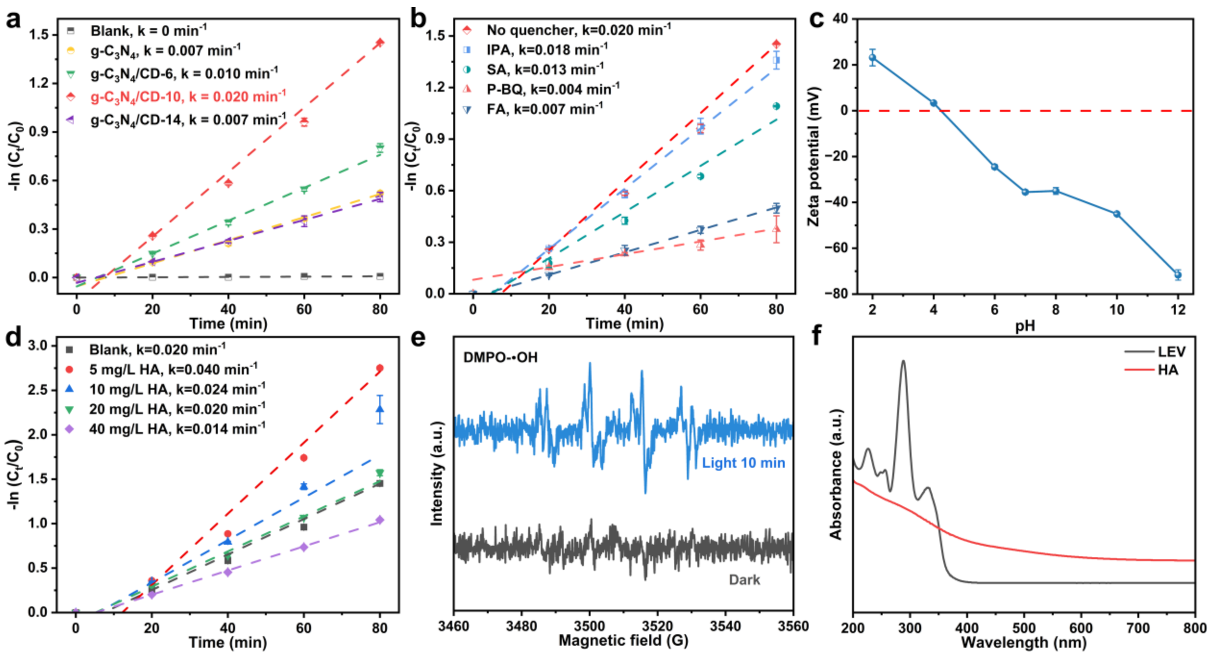

Figure S6. (a) First-order constants of different photocatalyst. (b) The rate constants of LEV degradation curves on g-C_3N_4/CD-10 with different scavengers under equivalent reaction conditions. (c) Zeta potential of g-C_3N_4/CD-10 at different pH values. (d) The corresponding rate constants of LEV degradation curves over g-C_3N_4/CD-10 alone and with different HA concentrations. (e) ESR signals for •OH of g-C_3N_4/CD-10 under 5 mg/L HA conditions. (f) UV-vis absorption spectra of LEV and HA.

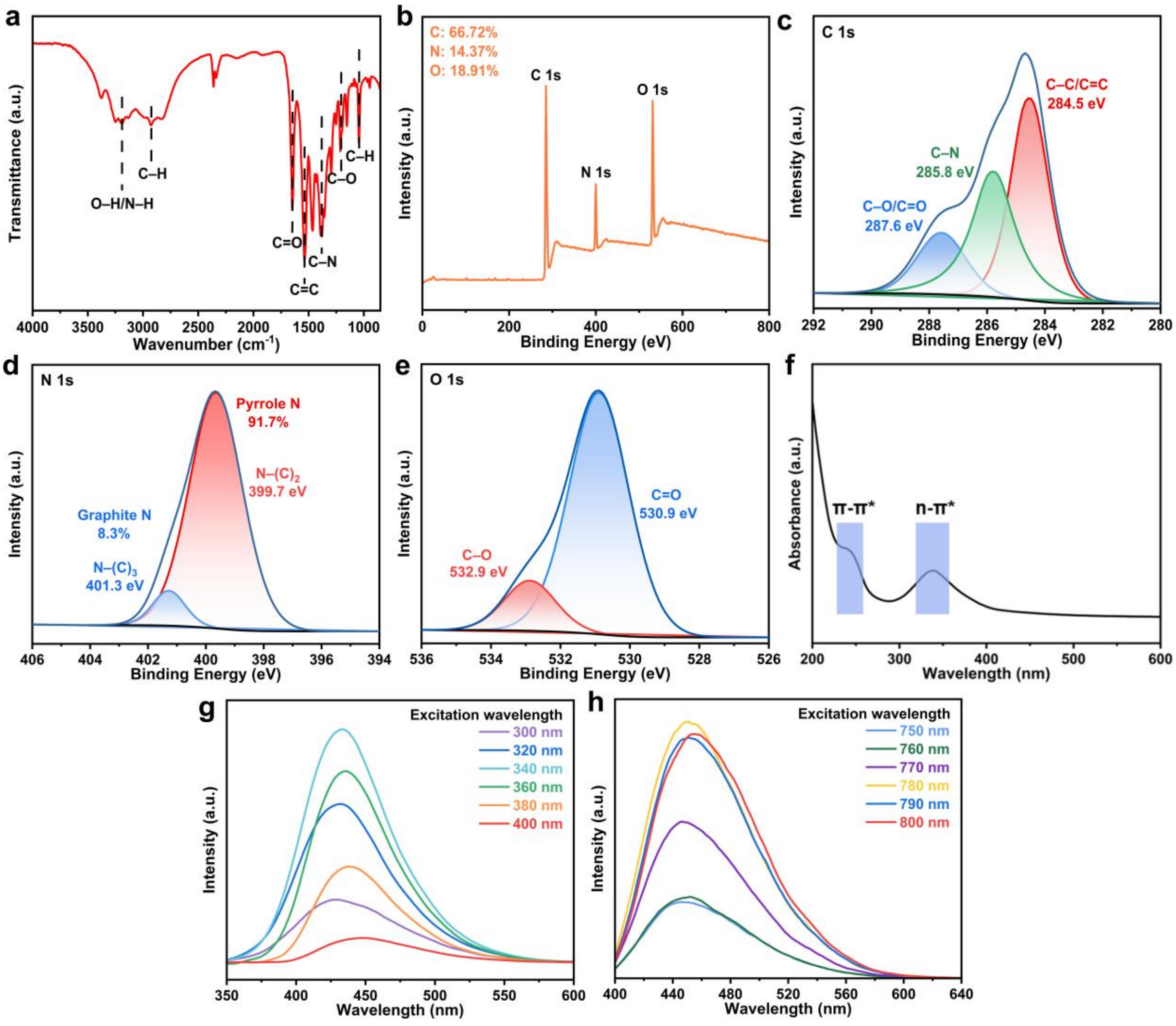

Figure S7. (a) FT-IR spectra, (b) XPS survey spectra, (c) high-resolution XPS C 1s spectra, (d) high-resolution XPS N 1s spectra, and (e) high-resolution XPS O 1s spectra of CD; (f) UV-vis absorption spectra (g) Excitation-emission PL spectra, and (h) up-conversion PL emission spectra of CD in aqueous solutions.

Supplementary Note 2:

The photophysical properties of CD were investigated by UV-vis absorption and emission spectroscopies. As shown in Figure S7f, the UV-vis absorption spectrum of CD exhibited two excitonic absorption bands at ca. 242 nm and 338 nm, with an optical absorption edge of about 600 nm, which could be excited in a wide wavelength range. The high-energy absorption peak below 300 nm belongs to the π-π* transition of the aromatic domains, while the low-energy broad absorption shoulder was attributed to the n–π* transition of the conjugated C–N in CD.

Upon excitation, a Gaussian emission band with peak maxima ranging from 427−447 nm has been recorded (Figure S7g) and assigned as originated from n–π* transition, mixing with some π–π* transition of the carbon core. Moreover, due to the excitation-dependent fluorescence behavior of carbon-based fluorescent materials, the PL maxima exhibit a redshift of about 20 nm, demonstrating a narrow particle size distribution of the CD, which is in agreement with the TEM results. The PL quantum yield (PLQY) of CD was measured at an excitation wavelength of 375 nm, and the yield was around 34.99%, suggesting that the synthesized CD possess a high fluorescence quantum yield (generally below 20% for CD).[21] It should be emphasized that the CD have excellent up-conversion PL properties. As shown in Figure S7h, the up-conversion spectra of CD could be regarded as an anti-Stokes transition, which was excited by long wavelength light (from 750 to 800 nm) giving up-conversion emissions in the range of 400−600 nm. This up-converted PL property of CD should be attributed to the multiphoton active process.[22] In summary, the synthesized CD can act as excellent near-infrared light harvesters, which enable it to be utilized as powerful energy conversion components in photocatalyst design and improve the light-harvesting capacity of the photocatalyst.

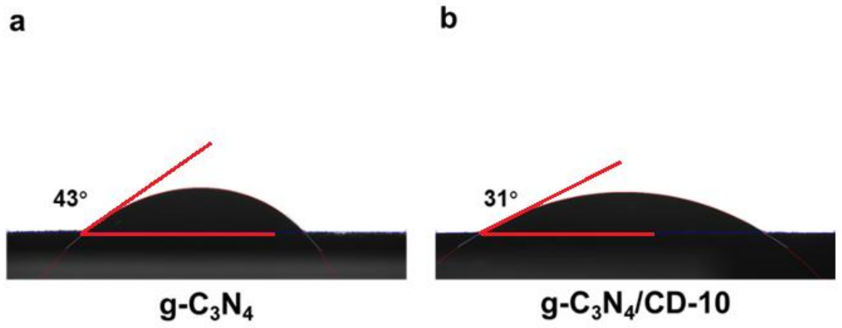

Figure S8. Contact angle of (a) g-C_3N_4 and (b) g-C_3N_4/CD-10.

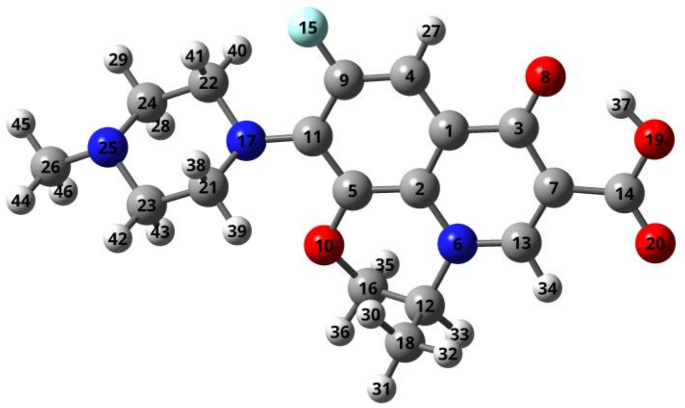

Figure S9. Labeling of LEV.

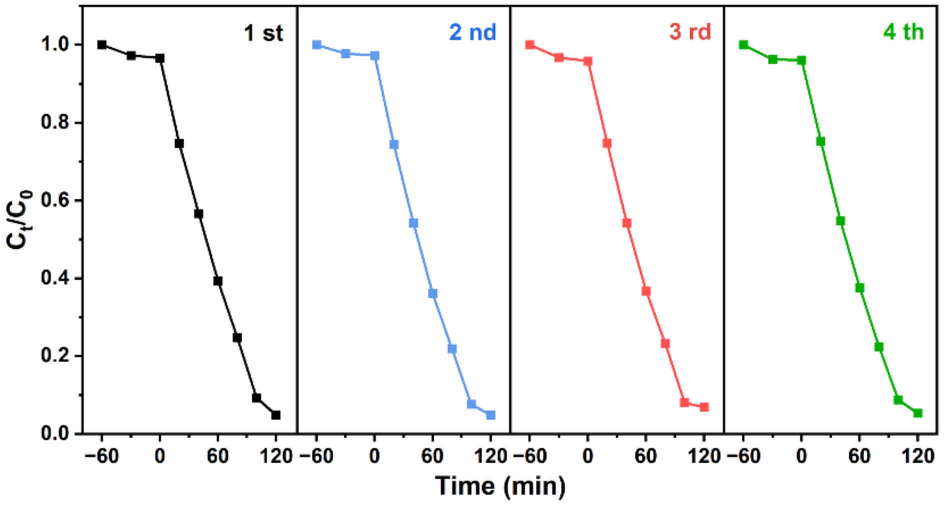

Figure S10. Recycling tests of LEV degradation over g-C_3N_4/CD-10.

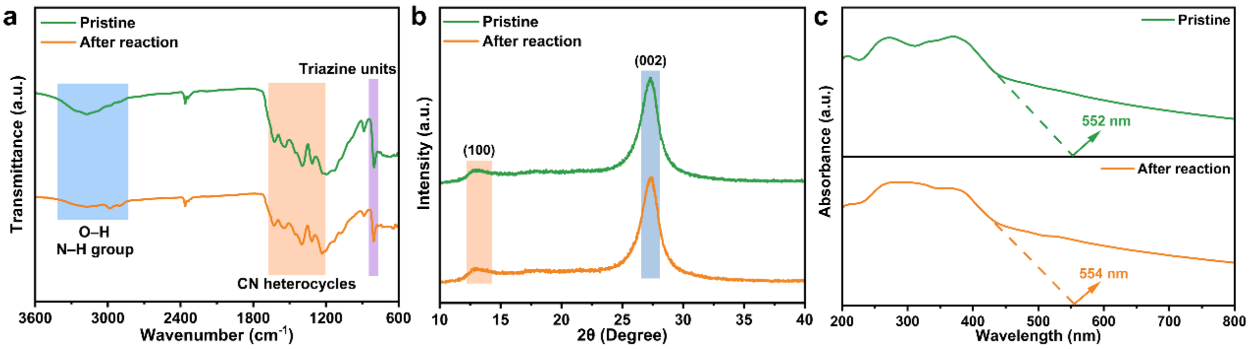

Figure S11. (a) FT-IR spectra, (b) XRD pattern spectra, and (c) UV–vis DRS spectra of g-C_3N_4/CD-10 before and after four reaction cycles.

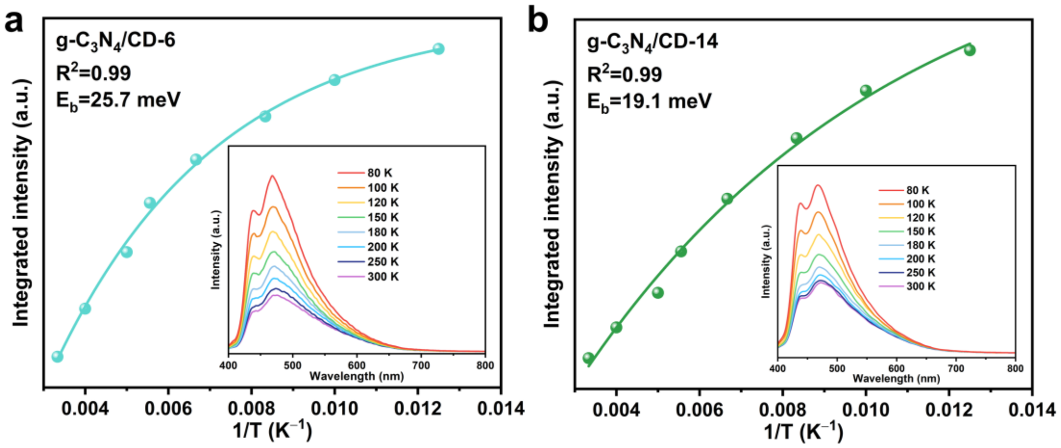

Figure S12. Integrated PL intensity as a function of temperature (insert: temperature-dependent PL spectra from 80 to 300 K) of (a) g-C_3N_4/CD-6, and (b) g-C_3N_4/CD-14.

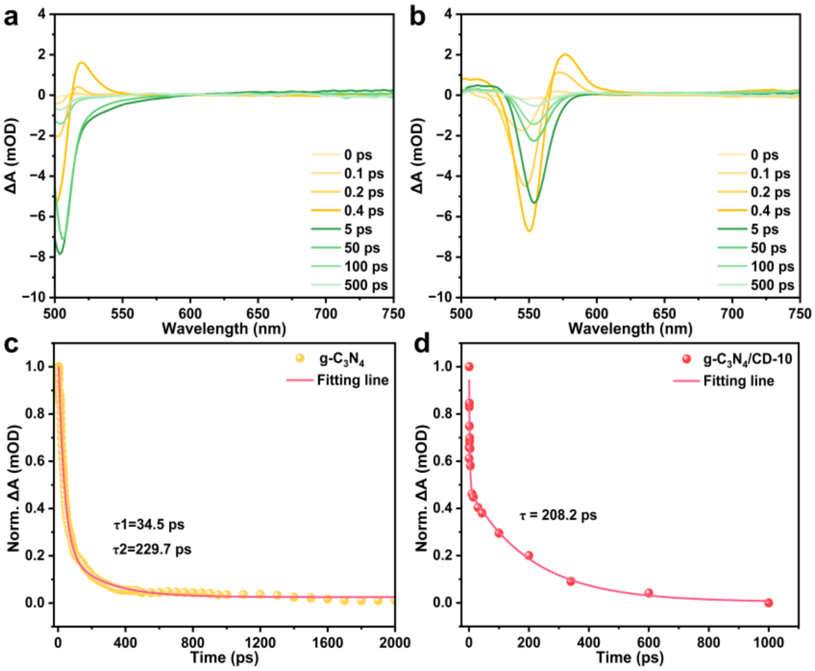

Figure S13. TAS measurements for (a) g-C_3N_4 and (b) g-C_3N_4/CD-10 under 365 nm excitation, and corresponding decay curves of normalized TAS of (c) g-C_3N_4 and (d) g-C_3N_4/CD-10 at 525 nm.

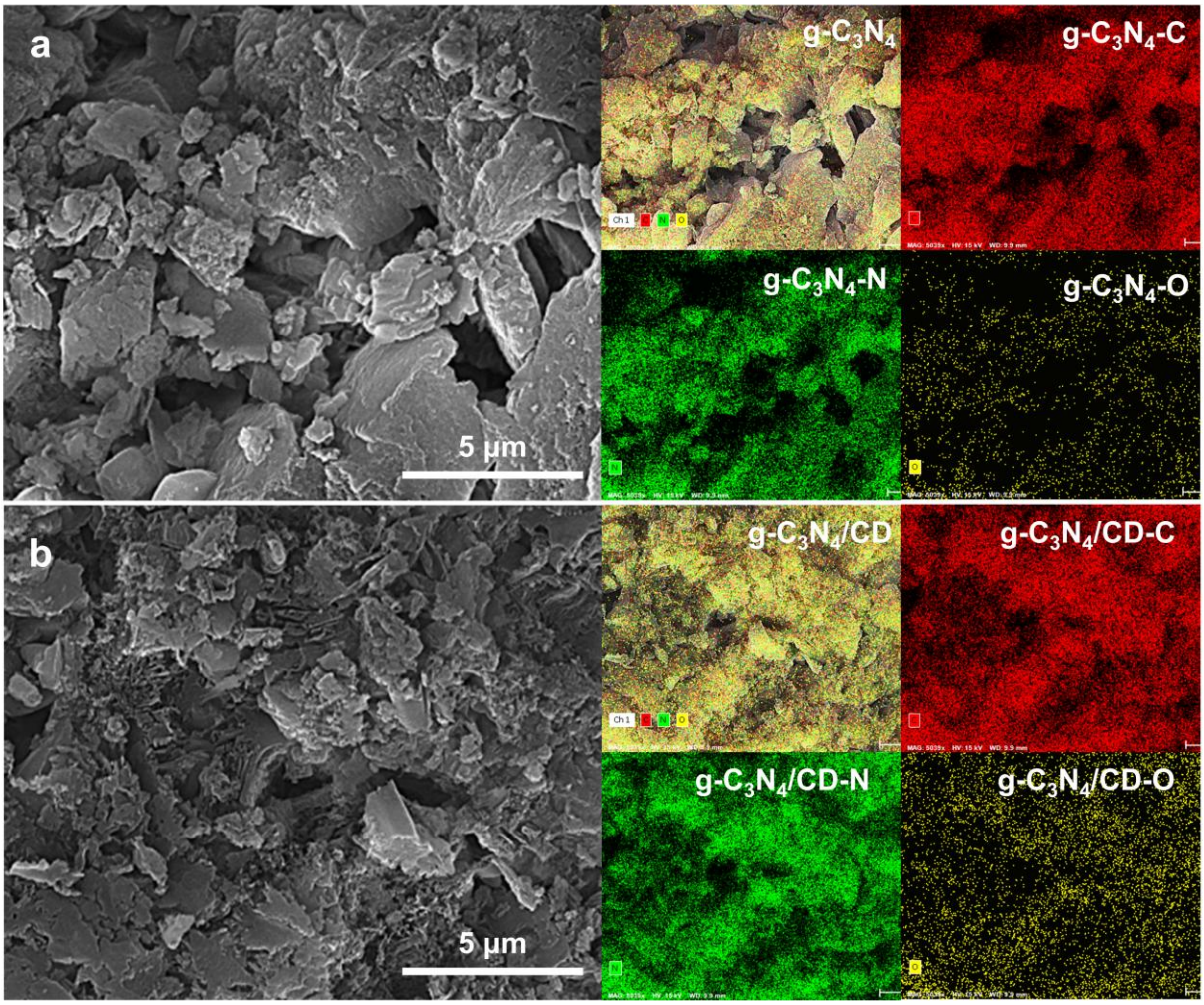

Figure S14. (a) SEM image of g-C_3N_4 and SEM/EDS mapping of C, N, and O elements in g-C_3N_4. (b) SEM image of g-C_3N_4/CD-10 and SEM/EDS mapping of C, N, and O elements in g-C_3N_4/CD-10.

Reference

[1] J. Zhang, X. Yuan, L. Jiang, Z. Wu, X. Chen, H. Wang, H. Wang, G. Zeng, *J. Colloid Interface Sci.* **2018**, *511*, 296–306.

[2] X. Liu, Y. Liu, S. Lu, Z. Wang, Y. Wang, G. Zhang, X. Guo, W. Guo, T. Zhang, B. Xi, *Chem. Eng. J.* **2020**, *385*, 123987.

[3] M. J. Frisch, G. W. Trucks, H. B. Schlegel, G. E. Scuseria, M. A. Robb, J. R. Cheeseman, G. Scalmani, V. Barone, G. A. Petersson, H. Nakatsuji, X. Li, M. Caricato, A. V. Marenich, J. Bloino, B. G. Janesko, R. Gomperts, B. Mennucci, H. P. Hratchian, J. V. Ortiz, A. F. Izmaylov, J. L. Sonnenberg, Williams, F. Ding, F. Lipparini, F. Egidi, J. Goings, B. Peng, A. Petrone, T. Henderson, D. Ranasinghe, V. G. Zakrzewski, J. Gao, N. Rega, G. Zheng, W. Liang, M. Hada, M. Ehara, K. Toyota, R. Fukuda, J. Hasegawa, M. Ishida, T. Nakajima, Y. Honda, O. Kitao, H. Nakai, T. Vreven, K. Throssell, J. A. Montgomery Jr., J. E. Peralta, F. Ogliaro, M. J. Bearpark, J. J. Heyd, E. N. Brothers, K. N. Kudin, V. N. Staroverov, T. A. Keith, R. Kobayashi, J. Normand, K. Raghavachari, A. P. Rendell, J. C. Burant, S. S. Iyengar, J. Tomasi, M. Cossi, J. M. Millam, M. Klene, C. Adamo, R. Cammi, J. W. Ochterski, R. L. Martin, K. Morokuma, O. Farkas, J. B. Foresman, D. J. Fox, Revision C.01 ed., *Gaussian 16*; Gaussian, Inc.: Wallingford CT, **2019**.

[4] a) A. D. Becke, *J. Chem. Phys.* **1993**, *98*, 5648-5652; b) C. T. Lee, W. T. Yang, R. G. Parr, *Phys. Rev. B* **1988**, *37*, 785-789; c) S. H. Vosko, L. Wilk, M. Nusair, *Can. J. Phys.* **1980**, *58*, 1200-1211; d) P. J. Stephens, F. J. Devlin, C. F. Chabalowski, M. J. Frisch, *J. Phys. Chem.* **1994**, *98*, 11623-11627.

[5] a) R. Ditchfield, W. J. Hehre, J. A. Pople, *J. Chem. Phys.* **1971**, *54*, 724-728; b) W. J. Hehre, Ditchfie.R, J. A. Pople, *J. Chem. Phys.* **1972**, *56*, 2257-2261; c) P. C. Hariharan, J. A. Pople, *Theor. Chim. Acta.* **1973**, *28*, 213-222; d) P. C. Hariharan, J. A. Pople, *Mol Phys* **1974**, *27*, 209-214; e) M. S. Gordon, *Chem. Phys. Lett.* **1980**, *76*, 163-168; f) M. M. Francl, W. J. Pietro, W. J. Hehre, J. S. Binkley, M. S. Gordon, D. J. Defrees, J. A. Pople, *J. Chem. Phys.* **1982**, *77*, 3654-3665; g) R. C. Binning, L. A. Curtiss, *J. Comput. Chem.* **1990**, *11*, 1206-1216; h) J. P. Blaudeau, M. P. McGrath, L. A. Curtiss, L. Radom, *J. Chem. Phys.* **1997**, *107*, 5016-5021; i) V. A. Rassolov, J. A. Pople, M. A. Ratner, T. L. Windus, *J. Chem. Phys.* **1998**, *109*, 1223-1229; j) V. A. Rassolov, M. A. Ratner, J. A. Pople, P. C. Redfern, L. A. Curtiss, *J. Comput. Chem.* **2001**, *22*, 976-984.

[6] A. V. Marenich, C. J. Cramer, D. G. Truhlar, *J. Phys. Chem. B* **2009**, *113*, 6378-6396.

[7] a) T. Lu, F. Chen, *J. Comput. Chem.* **2012**, *33*, 580-592; b) J. Zhang, T. Lu, *Phys. Chem. Chem. Phys.* **2021**, *23*, 20323-20328.

[8] Y. Zhao, D. G. Truhlar, *Theor. Chem. Acc.* **2008**, *120*, 215-241.

[9] a) R. Bauernschmitt, R. Ahlrichs, *Chem. Phys. Lett.* **1996**, *256*, 454-464; b) M. E. Casida, C. Jamorski, K. C. Casida, D. R. Salahub, *J. Chem. Phys.* **1998**, *108*, 4439-4449; c) R. E. Stratmann, G. E. Scuseria, M. J. Frisch, *J. Chem. Phys.* **1998**, *109*, 8218-8224.

[10] S. Hirata, M. Head-Gordon, *Chem. Phys. Lett.* **1999**, *314*, 291-299.

[11] T. Yanai, D. P. Tew, N. C. Handy, *Chem. Phys. Lett.* **2004**, *393*, 51-57.

[12] Z. Liu, T. Lu, Q. Chen, *Carbon* **2020**, *165*, 461-467.

[13] a) X. Zhang, Y. Zhang, X. Jia, N. Zhang, R. Xia, X. Zhang, Z. Wang, M. Yu, *Sep. Purif. Technol.* **2021**, *268*, 118691; b) F. Hasanvandian, A. Shokri, M. Moradi, B. Kakavandi, S. Rahman Setayesh, *J. Hazard. Mater.* **2022**, *423*, 127090. c) X. Zhang, Y. Liu, M. Ren, G. Yang, L. Qin, Y. Guo, J. Meng, *Chem. Eng. J.* **2022**, *433*, 134551; d) C. Zhang, L. Lin, M. Zhou, Y. Wang, S. Xu, X. Chen, Z. Li, *Chem. Eng. J.* **2024**, *495*, 153563; e) H. Wei, F. Meng, W. Yu, J. Li, H. Zhang, *Sep. Purif. Technol.* **2023**, *318*, 123940; f) L. Zhu, D. Shen, H. Zhang, K. H. Luo, C. Li, *J. Hazard. Mater.* **2023**, *446*, 130663; g) X. Yang, M. D. Hesami, E. Nazemipool, A. Bahadoran, M. Al-Bahrani, B. Azizi, *Sep. Purif. Technol.* **2022**, *301*, 122005; h) L. Li, C. G. Niu, H. Guo, J. Wang, M. Ruan, L. Zhang, C. Liang, H. Y. Liu, Y. Y. Yang, *Chem. Eng. J.* **2020**, *383*, 123192; i) C. K. Tsai, Y. C. Lee, T. T. Nguyen, J. J. Horng, *Chemosphere* **2022**, *298*, 134285; j) G. Dong, W. Chi, D. f. Chai, Z. Zhang, J. Li, M. Zhao, W. Zhang, J. Lv, S. Chen, *Appl. Surf. Sci.* **2023**, *619*, 156732; k) J. Nie, X. Yu, Z. Liu, Y. Wei, J. Zhang, N. Zhao, Z. Yu, B. Yao, *Appl. Surf. Sci.* **2022**, *576*, 151842; l) T. Cao, J. Xu, M. Chen, *Sep. Purif. Technol.* **2022**, *291*, 120896; m) C. Qin, Y. Yang, X. Wu, L. Chen, Z. Liu, L. Tang, L. Lyu, D. Huang, D. Wang, C. Zhang, X. Yuan, W. Liu, H. Wang, *Nat. Commun.* **2023**, *14* (1), 6740; n) S. Li, C. Wang, M. Cai, F. Yang, Y. Liu, J. Chen, P. Zhang, X. Li, X. Chen, *Chem. Eng. J.* **2022**, *428*, 131158;

[14] Z. Gao, Z. Lin, X.-m. Chen, Z. Lai, Z. Huang, *Sens. Actuators, B* **2016**, *222*, 965-971.

[15] L. Yan, L. Xie, X. L. Wu, M. Qian, J. Chen, Y. Zhong, Y. Hu, *Carbon Energy* **2021**, *3*, 856-865.

[16] S. Zhang, M. Gao, Y. Zhai, J. Wen, J. Yu, T. He, Z. Kang, S. Lu, *J. Colloid Interface Sci.* **2022**, *622*, 662-674.

[17] G. Peng, L. Xing, J. Barrio, M. Volokh, M. Shalom, *Angew. Chem. Int. Ed.* **2018**, *57*, 1186-1192.

[18] J. Fu, Q. Xu, J. Low, C. Jiang, J. Yu, *Appl. Catal., B* **2019**, *243*, 556-565.

[19] J. Wu, N. Li, H. B. Fang, X. Li, Y. Z. Zheng, X. Tao, *Chem. Eng. J.* **2019**, *358*, 20-29.

[20] J. Huang, Y. Cao, H. Wang, H. Yu, F. Peng, H. Zou, Z. Liu, *Chem. Eng. J.* **2019**, *373*, 687-699.

[21] H. Ding, J. S. Wei, N. Zhong, Q. Y. Gao, H. M. Xiong, *Langmuir* **2017**, *33*, 12635-12642.

[22] J. Shen, Y. Zhu, C. Chen, X. Yang, C. Li, *Chem. Commun.* **2011**, *47*, 2580-2582.
